# Broad anti-pathogen potential of DEAD box RNA helicase eIF4A-targeting rocaglates

**DOI:** 10.1038/s41598-023-35765-6

**Published:** 2023-06-08

**Authors:** Wiebke Obermann, Mohammad Farhan Darin Azri, Leonie Konopka, Nina Schmidt, Francesca Magari, Julian Sherman, Liliana M. R. Silva, Carlos Hermosilla, Andreas H. Ludewig, Hicham Houhou, Simone Haeberlein, Mona Yiting Luo, Irina Häcker, Marc F. Schetelig, Christoph G. Grevelding, Frank C. Schroeder, Gilbert Sei Kung Lau, Anja Taubert, Ana Rodriguez, Andreas Heine, Tiong Chia Yeo, Arnold Grünweller, Gaspar Taroncher-Oldenburg

**Affiliations:** 1 Institute of Pharmaceutical Chemistry, Philipps University Marburg, Marburg, Germany; 2 Sarawak Biodiversity Centre, Kuching, Sarawak Malaysia; 3 Department of Microbiology, New York University Grossman School of Medicine, New York, NY USA; 4 Institute of Parasitology, Faculty of Veterinary Medicine, Justus Liebig University Giessen, Giessen, Germany; 5 Boyce Thompson Institute, Department of Chemistry and Chemical Biology, Cornell University, Ithaca, NY USA; 6 Institute for Insect Biotechnology, Justus Liebig University Giessen, Giessen, Germany; 7 Gaspar Taroncher Consulting, Philadelphia, PA USA

**Keywords:** Biomarkers, Target validation, Evolutionary genetics, Drug discovery, Evolution, Biochemistry, Isoenzymes

## Abstract

Inhibition of eukaryotic initiation factor 4A has been proposed as a strategy to fight pathogens. Rocaglates exhibit the highest specificities among eIF4A inhibitors, but their anti-pathogenic potential has not been comprehensively assessed across eukaryotes. In silico analysis of the substitution patterns of six eIF4A1 aa residues critical to rocaglate binding, uncovered 35 variants. Molecular docking of eIF4A:RNA:rocaglate complexes, and in vitro thermal shift assays with select recombinantly expressed eIF4A variants, revealed that sensitivity correlated with low inferred binding energies and high melting temperature shifts. In vitro testing with silvestrol validated predicted resistance in *Caenorhabditis* *elegans* and *Leishmania* *amazonensis* and predicted sensitivity in *Aedes* sp., *Schistosoma* *mansoni*, *Trypanosoma* *brucei*, *Plasmodium* *falciparum*, and *Toxoplasma* *gondii*. Our analysis further revealed the possibility of targeting important insect, plant, animal, and human pathogens with rocaglates. Finally, our findings might help design novel synthetic rocaglate derivatives or alternative eIF4A inhibitors to fight pathogens.

## Introduction

Targeting eukaryotic translation has emerged as a potential strategy to combat pathogens^1–4^. Of the three steps that constitute translation—initiation, elongation and termination, initiation has garnered particular interest as a target due to the many factors involved and to its rate limiting effect on translation overall^5–7^.

The highly conserved eukaryotic translation initiation factor 4A (eIF4A), an ATP-dependent DEAD-box RNA helicase, plays an essential role in the initiation of translation^8,9^. There are two isoforms of eIF4A, eIF4A1 and eIF4A2, with equivalent biochemical functions and a sequence identity of 90–95%^10,11^. Expression of both isoforms differs substantially, eIF4A1 being present in almost all tissues during active cell growth and eIF4A2 mainly in organs with low proliferation rates^12^.

Rocaglates, a class of plant-derived flavaglines containing a cyclopenta[*b*]benzofuran structure, are among the most potent and specific eIF4A inhibitors known^13,14^ (Fig. S1). Over 200 natural and synthetic rocaglates have been described since rocaglamide A (RocA) was first isolated from Asian mahogany (*Aglaia* sp.), the only genus known to produce rocaglates^15–18^. Rocaglates clamp eIF4A:RNA complexes containing RNA strands with stable secondary structures such as stem-loops or G-quadruplexes and also polypurine stretches in the 5’UTR, all associated with subclasses of mRNAs including proto-oncogenes and viral mRNAs^19–21^. Such mRNAs are preferentially processed by eIF4A and are often associated with proliferating cells and translational regulation^22–24^. The preference of eIF4A to unwind mRNAs with stable secondary structures in their 5’UTRs, and the avidity of rocaglates for the resulting eIF4A:RNA complexes, makes this a potentially viable approach to fight pathogens due to low toxicities for humans and animals^25^.

In vivo studies have demonstrated the therapeutic potential of rocaglates in cancer. The synthetic analog zotatifin is currently undergoing a Phase 1/2 clinical study for solid tumors^26^ and a dose-escalating Phase 1 clinical study for COVID-19, indicating its potential for host-targeted antiviral activity^27^. Multiple other studies have shown the effectiveness of rocaglates in preventing the replication of multiple RNA viruses, supporting the potential of rocaglates as pan-antivirals^28–33^.

The eIF4A-dependent anti-pathogenic potential of rocaglates has been shown for *Plasmodium* *falciparum* and *P.* *berghei*, *Candida* *auris*, and several other eukaryotic pathogens^3,34^ (see Table S1). Other pathogens are resistant to rocaglates, e.g., *Entamoeba* *histolytica* and *Leishmania* *donovani*^35,36^ (see Table S1).

Rocaglates bind to eIF4A:RNA complexes, leading to a stable ternary complex that prevents enzymatic mRNA unwinding^37,38^. Select aa within the rocaglate/RNA-binding pocket—human eIF4A1 positions 158, 159, 163, 192, 195, 199—are critical to the rocaglate clamping mechanism^38–40^. The crystal structure of human eIF4A1 in complex with RocA, polypurine RNA, and a non-hydrolyzable ATP analogue, shows that rocaglates reversibly clamp mRNAs to eIF4A through π–π-stacking interactions between the rocaglates’ A and B phenyl rings and two purines in the RNA, and between the rocaglates’ C phenyl ring and the amino acid residue at position 163^38^. The eIF4A sequences of rocaglate-producing *Aglaia* spp. exhibit resistant substitutions that prevent complex formation at both aa 163 (Phe to Leu) and aa 199 (Ile to Met), and a fungal parasite of *Aglaia*, *Ophiocordyceps* sp. BRM1, exhibits another resistance conferring substitution at aa 163 (Phe to Gly)^38,41^.

Here, we present an in silico analysis of eIF4A sequences, together with a biochemical analysis of representative sequences and in vitro studies with eukaryotic microorganisms containing previously untested variants of eIF4A, providing a first comprehensive picture of the anti-pathogenic potential of rocaglates. The results reveal potential evolutionary scenarios for rocaglate resistance, and provide insights into structure–activity relationships that could inform the design of next-generation rocaglates or other eIF4A inhibitors.

## Results

### Global eIF4A sequence analysis reveals limited diversity of rocaglate-interacting aa patterns in the RNA-binding pocket

A global GenBank (https://www.ncbi.nlm.nih.gov/genbank/) search for eIF4A sequences produced 365 unique eIF4A1 and eIF4A2 protein sequences—78 protist, 80 fungal, 49 plant, and 158 animal (Table S2). Of these, 162 corresponded to pathogens of protist (55), fungal (53), or animal (54) origin. To determine the potential interactions of these eIF4A proteins with rocaglates, we analyzed the substitution patterns of six aa residues (human positions 158, 159, 163, 192, 195, 199) located between motifs Ib and III of eIF4A^38,39,42–44^ (Fig. S2). The aa patterns known to be associated with sensitivity or resistance to rocaglates are listed in Table S1.

Our analysis uncovered 35 aa patterns. Four of them—T158, P159, Y163, F192, Q195, V199; TPFFQI; TPFFQV; TPYFQI—accounted for 63% of all known eIF4A sequences and were present in the four major eukaryotic lineages (Fig. S3). Over half of the aa patterns (24/35) were present in only one lineage. The pattern TPLFQM was present in all rocaglate-producing *Aglaia* spp., including two novel species reported here, *Aglaia* *stellatopilosa* and *Aglaia* *glabriflora* (Fig. S2; Accession numbers ON844099 and ON844100, respectively). The pattern TPGFQI was also present in only one species: the fungal parasite of *Aglaia* spp., *Ophiocordyceps*.

**Figure 1:**
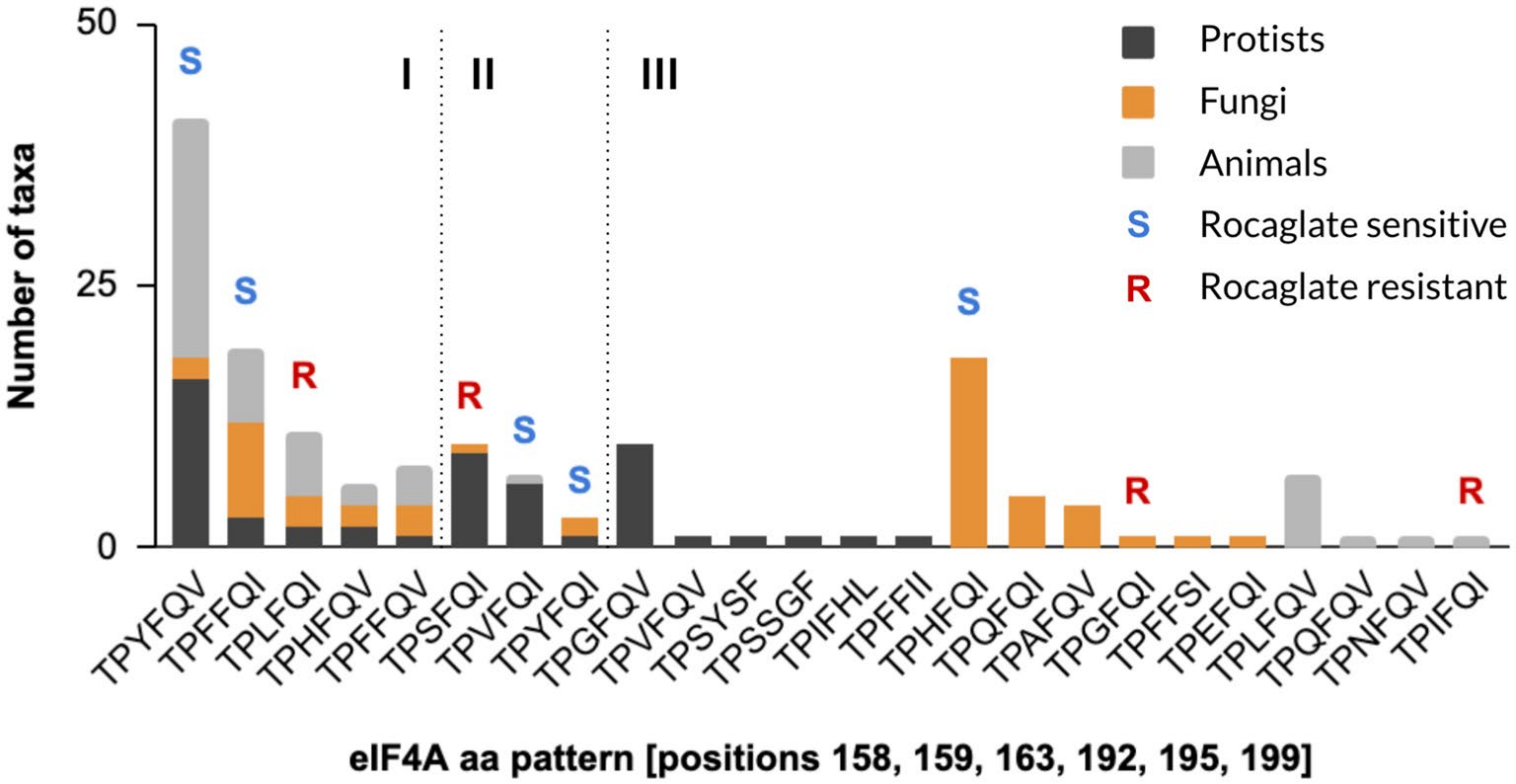
Representation of patterns of aa critical for rocaglate-binding in known eIF4A proteins across the three main groups of eukaryotic pathogens. A comprehensive analysis of known eIF4A proteins encoded by pathogens revealed 24 patterns of aa at positions 158, 159, 163, 192, 195, and 199 (human eIF4A1 numbering). Five aa patterns were present in all three groups of eukaryotes (I), three patterns were present in two groups (II) and sixteen patterns were present in only one group (III). Patterns known to provide natural sensitivity or resistance to rocaglates could be found in all three groups.

Among the 162 pathogens analyzed, we identified 24 aa patterns. The protists contained the highest diversity (14) followed by fungi (13) and animals (9) (Table S2). Five patterns—TPYFQV, TPFFQI, TPLFQI, TPHFQV, TPFFQV—accounted for 53% of all pathogen eIF4A sequences (Fig. 1). Two thirds of the aa patterns (16/24) were present in only one lineage. Importantly, four aa patterns associated with sensitivity to rocaglates—TPYFQV, TPFFQI, TPYFQI, TPHFQI—could be detected in 50% of the pathogens analyzed (Fig. 1). Another 14% of the sequences (22/162) exhibited aa patterns—TPLFQI, TPSFQI, TPGFQI—previously associated with resistance to rocaglates (Table S1).

Pathogens and vectors potentially sensitive to rocaglates include *Trichuris* *trichiura*, *Glossina* *morsitans*, *Aspergillus* *niger*, and *Cryptosporidium* sp.; potentially resistant pathogens include *Paragonimus* *westermani*, *Blumeria* *graminis*, and *Leishmania* sp. (Table S2).

### Substitution tolerance analysis of rocaglate-interacting aa patterns reveals dichotomy between highly conserved and highly variable residues

**Figure 2:**
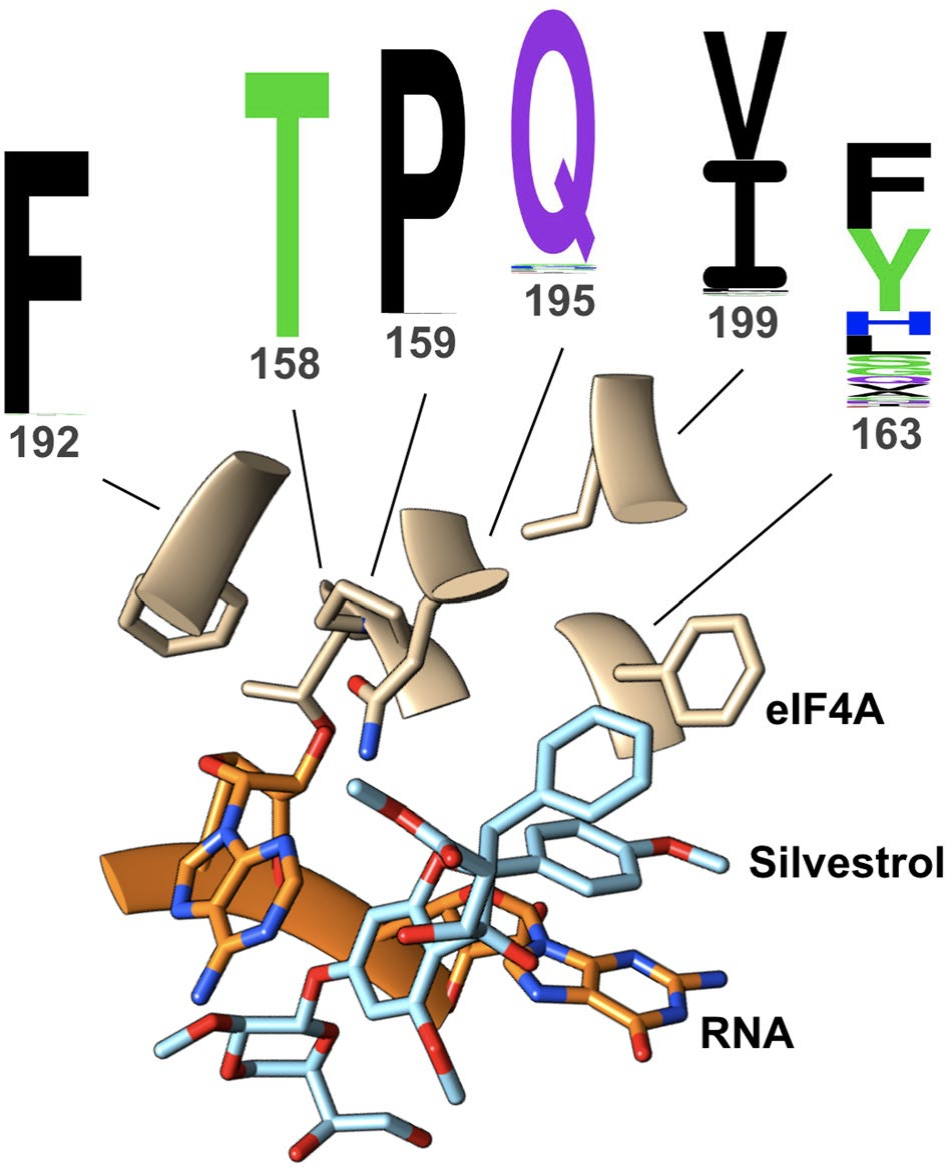
Amino acid substitution profiles at six positions known to be critical for rocaglate binding. Six aa residues—158, 159, 163, 192, 195, 199—in the eIF4A RNA-binding pocket have been shown to be critical for rocaglate binding. Natural substitution tolerances at each of the six residues across the 365 eIF4A sequences analyzed here revealed four highly conserved positions—158, 159, 192, 195—and two variable positions—163 and 199. Of these, the latter shows a quasi bimodal frequency distribution (I or V), while the former shows a more promiscuous frequency distribution, with 14 different aa being tolerated in that position. The structure depicted above corresponds to the aa pattern TPFFQI; the rocaglate shown interacting with the eIF4A:RNA complex is silvestrol. The sequence logos were generated with Seq2Logo—2.0^50^.

Substitution tolerances at the six rocaglate-binding positions were markedly different, with four positions—158, 159, 192, 195—being highly conserved and the two other positions—163, 199—showing different degrees of variation (Fig. 2). Amino acid positions 158, 192, and 195 are involved in RNA binding, and position 159, while itself not directly involved in RNA binding, is flanked by three RNA-binding residues—158, 160, and 161 (Fig. S2)^42^. Position 163 showed a high level of tolerance for substitutions, with 14 different aa filling the position across all eIF4As surveyed. The six aa not detected in position 163—positively-charged basic [Arg, Lys], nonpolar aliphatic [Met, Pro], nonpolar aromatic [Trp], and polar [Thr]—have been associated with destabilization of α-helices [Pro] or modulation of protein interactions with nucleic acids [Arg]^45–47^. Position 199, part of an α-helix situated between motifs II and III of eIF4A, exhibited a quasi bimodal tolerance for Ile or Val, two structurally similar aliphatic aa of equivalent hydrophobicity. At the codon level, the switch from Ile to Val only requires the first nucleotide to switch from adenine to guanine (AUA, AUU, or AUC to GUA, GUU, or GUC), and the switch from Ile to Met, only observed in *Aglaia*, requires a change of the third nucleotide, the ‘wobble position’, in any of the Ile codons to guanine (AUA, AUU, or AUC to AUG)^48^.

The conservation of residues T158, P159, F192, and Q195, and the limited tolerance for Val and Ile at position 199, confirmed position 163 as the key determinant of sensitivity to rocaglates^38,39^. Several of the substitutions at position 163—Glu, Asp, His, Phe, Tyr—can establish either π–π-stacking or hydrophobic interactions, rendering the variants sensitive to rocaglates^49^.

Substitutions at position 163 were not uniform across lineages. In protists, 29% of the sequences had Tyr in position 163, followed by Ser (15%), Phe (14%), Gly (14%), and Val (12%). In fungi the distribution was His (36%), Phe (24%), Gln (12%), and Ala (10%). In plants, 73% of the eIF4A sequences had Phe in position 163 and 10% Tyr, while in animals the proportion was 51% Tyr and 34% Phe (Table S2; Fig. S3).

### Evolutionary analysis of eIF4A aa patterns associated with rocaglate sensitivity suggests emergence of resistance is serendipitous

**Figure 3:**
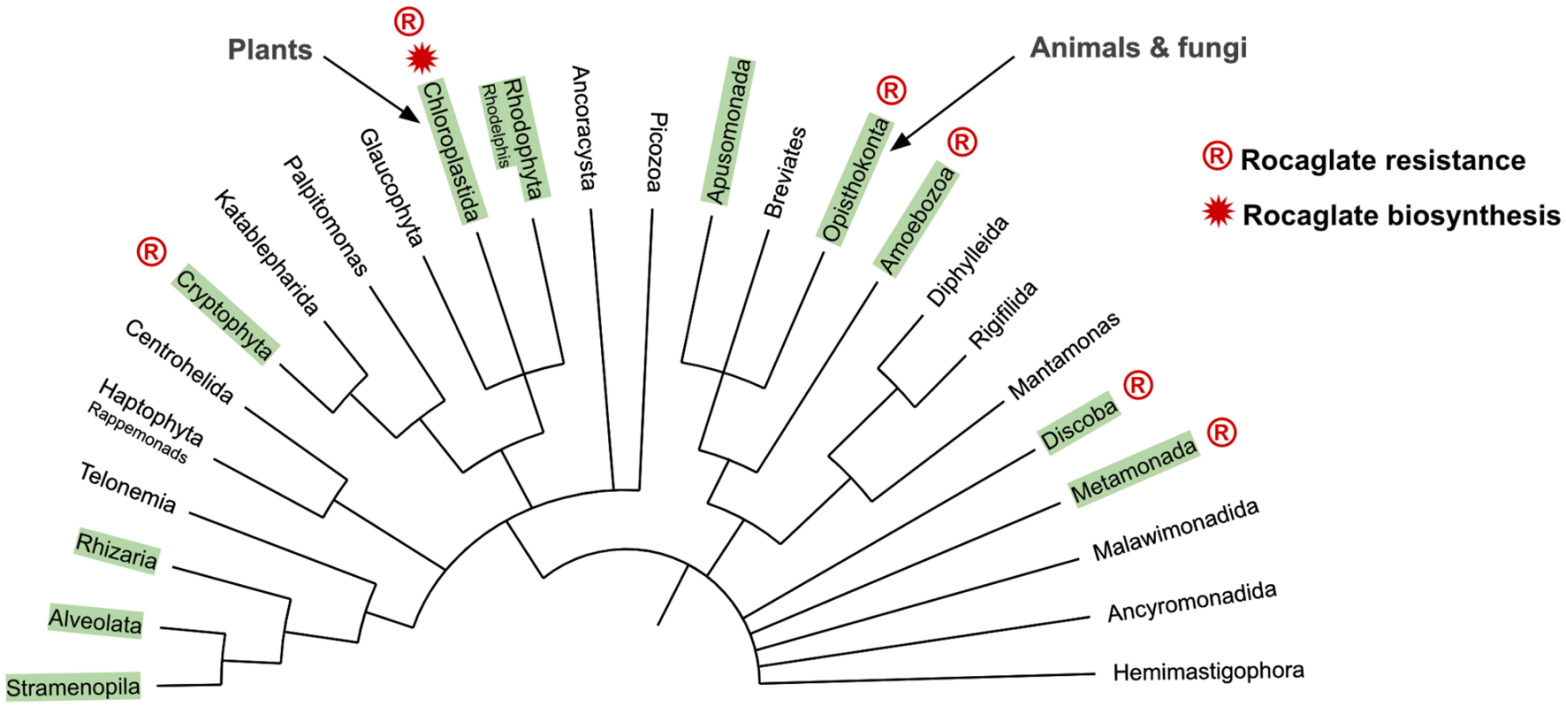
Distribution of eIF4A rocaglate resistance on the eToL. Green boxes denote clades represented in our eIF4A analysis. Rocaglate resistant variants can be found in deep rooted as well as more recently evolved clades. Several variants are exclusive to one clade while others are distributed more generally.

To gain an evolutionary understanding of the emergence of resistance to rocaglates, we projected the 365 sequences onto the eukaryotic tree of life (eToL) (Fig. 3)^51^. Of the eleven clades represented, six, including deep-rooted branches such as discoba (e.g., *Leishmania* sp.) and metamonada (e.g., *Giardia* sp.), contained organisms resistant to rocaglates.

Only plants within the genus *Aglaia*, which like all higher plants emerged much later in evolution, produce rocaglates. This asynchrony in the emergence of rocaglate synthesis and eIF4A resistance to it suggests ‘rocaglate resistance’ cannot be driven by exposure to rocaglates but be an unintended result of eIF4A sequence diversification. An exception is the *Aglaia* sp. parasitic fungus *Ophiocordyceps* sp. BRM1, which could have developed resistance through direct exposure^41^. In *Aglaia*, resistance could have emerged as the ability to synthesize rocaglates evolved. The rocaglate-resistant aa pattern of the *Aglaia* eIF4A, TPLFQM, contains the most common ‘resistant’ substitution at position 163, Leu.

### Subgroup of organisms contains eIF4A isoforms with divergent rocaglate-associated aa patterns

We uncovered several organisms whose eIF4A isoforms, eIF4A1 and eIF4A2, contain divergent rocaglate-associated aa patterns (Table S3). Among protists there were no divergent patterns except between the two eIF4A isoforms of *Thecamonas* *trahens*: the archetypal rocaglate-sensitive TPFFQI and a pattern containing three substitutions, TPLFAV. The L163 points toward potential resistance to rocaglates, but no in vitro or in vivo confirmation exists. Among fungi, five species belonging to five different genera exhibited divergent isoforms. All isoforms switched between His and Ala at position 163, and in four cases between Ile and Val in position 199; the fifth pair had a Val at this position. Three species—*Neurospora* *crassa*, *Purpureocillium* *lilacinum*, and *Diplocarpon* *rosae*—are known pathogens. The effect of the H163A substitution on resistance is not known. Among plants, the six genera with divergent aa patterns exhibited several substitution patterns. Three isoform pairs switched between a Cys and a Phe at position 163—requiring just a point mutation of the second nucleotide of the corresponding codon from guanine to uracil (UGC or UGU to UUC or UUU)—accompanied by a switch from a Val to Ile in position 199. Another isoform pair switched between Ile and Val at position 199 but retained the F163 in both isoforms, and the microalga *Raphidocelis* *subcapitata* had isoforms with a unique substitution pattern: Y163N and L199C. Finally, all species of rocaglate-producing *Aglaia* exhibited a unique combination of resistant isoforms, TPLFQM and TPLFQI.

Animals exhibited the largest number of divergent eIF4A isoforms. Of 14 pairs, 11 had likely neutral substitutions—F163Y and I199V—and five had an F163L substitution rendering one of the isoforms resistant. All diverging aa patterns with a resistant/sensitive dichotomy were found in pathogens—*Trichuris* *trichiura*, *Schistocephalus* *solidus*, *Hymenolepis* *microstoma*, and *Schistosoma* *mansoni*.

### Comparative analysis of RNA helicase activities of representative eIF4A variants suggests evolutionary convergence toward optimal enzyme performance independent of rocaglate resistance

The overlap among rocaglate- and RNA-interacting residues, raises the question of how particular substitutions could affect the RNA helicase activity of eIF4A. Single mutations in positions 159, 163, and 195 have been reported to have no or only little effect on RNA helicase activity^39^. To systematically determine the effect of aa substitutions at positions 163 and 199 on the helicase activity, we generated 17 mutant eIF4A proteins with single and double substitutions in a human eIF4A1 background (Table S4). Five variants contained non-natural substitutions to test potential evolutionary constraints.

**Figure 4:**
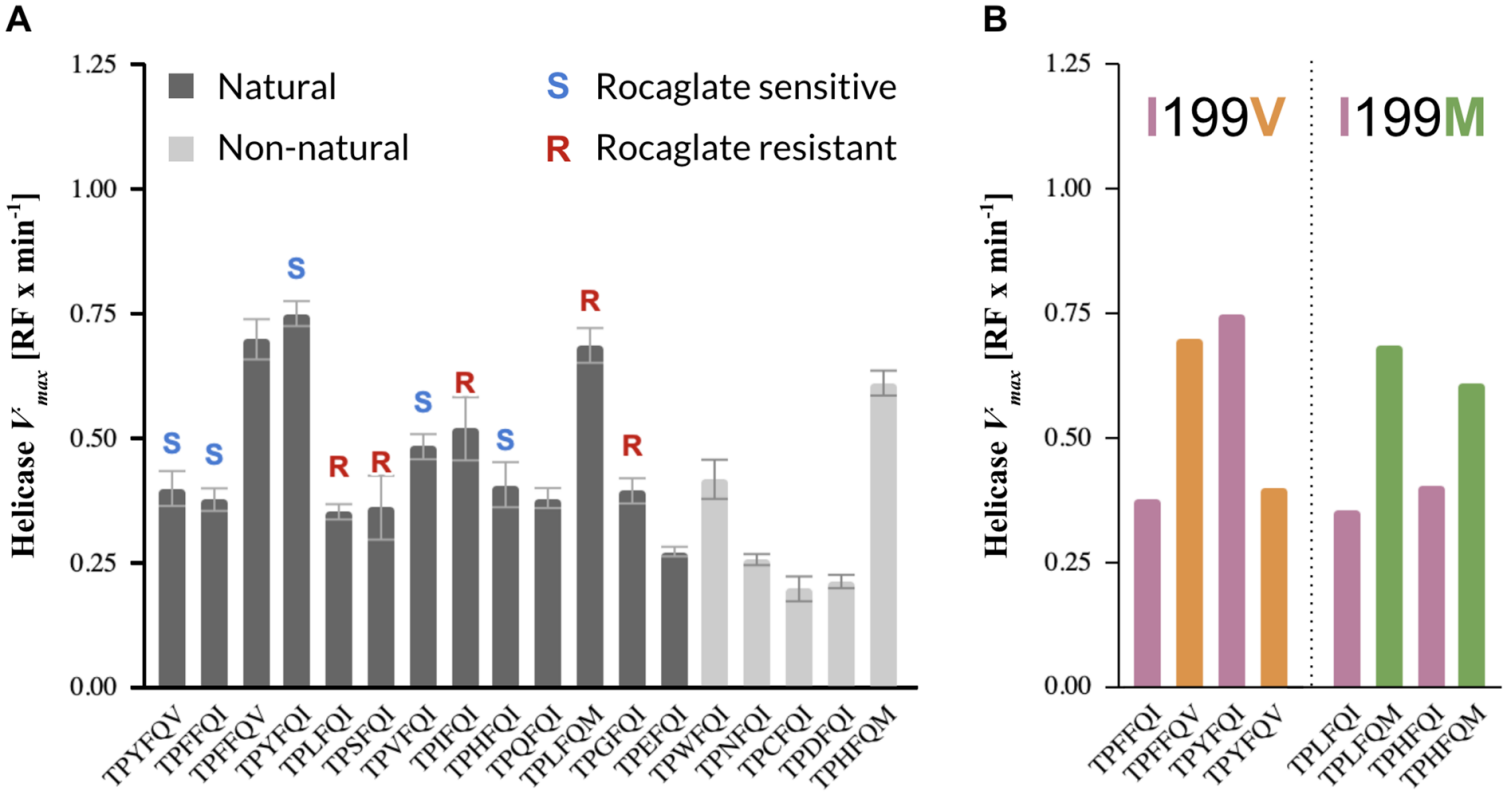
RNA helicase activities of eIF4A mutant proteins containing select natural and non-natural rocaglate-associated aa patterns in a human whole protein background. (**A**) Helicase *V*_*max*_ of the wild-type human eIF4A1 containing the rocaglate-binding pattern TPFFQI and 17 variants of the human eIF4A1 protein (Table S4), including one expressing the *Aglaia* sp. aa pattern TPLFQM. All natural and non-natural aa patterns analyzed exhibited *V*_*max*_ within a narrow range, indicating that the helicase activity is maintained. (**B**) Side-by-side *V*_*max*_ comparison of mutant pairs differing only in the aa residue at position 199 of the eIF4A protein. The two substitutions analyzed, I199V and I199M, are the only natural substitutions at this position revealed by our global eIF4A sequence survey (*RF* relative fluorescence; error bars indicate mean standard error from three technical replicates; error bars removed in (**B**) for clarity).

Comparison of the RNA helicase activity profiles revealed a tight distribution of *V*_*max*_ across variants (Fig. 4A). The five non-natural patterns showed a similar range of *V*_*max*_ to the natural patterns. Three of the natural substitution patterns—TPFFQV, TPYFQI, and TPLFQM—exhibited the highest helicase activities. Among the non-natural patterns, a mutant derived from the *Aglaia* sp. pattern TPLFQM, TPHFQM, stood out for exhibiting a *V*_*max*_ more similar to that of the three high *V*_*max*_ natural patterns.

Two naturally occurring substitutions at position 199—I199V and I199M—showed a modulating effect on the helicase activity (Fig. 4B). The switch from Ile to Val enhanced or reduced *V*_*max*_ depending on the aa at position 163; the switch from Ile to Met resulted in an equivalent enhancement of the corresponding *V*_*max*_, regardless of the aa at position 163. The similarity in effect size, directionality, or both, of these select single point mutations at position 199 on the *V*_*max*_, and the fact that it was within the range of natural *V*_*max*_ determined for natural eIF4A variants, suggests that substitution tolerance is determined by the efficiency of the helicase.

The tolerance for substitutions at position 163 suggested that residues of diverse physicochemical characteristics do not substantially affect helicase activity. All variants analyzed here were evaluated within the same human protein framework to remove potential structural confounders. Ultimately, each of the patterns would have to be evaluated within its natural scaffold to determine the real *V*_*max*_ of each eIF4A variant.

### Comparative thermal shift analysis of eIF4A:RNA:rocaglate complexes reveals direct correlation between rocaglate sensitivity and complex stability

Clamping of the mRNA to eIF4A requires π–π-stacking interactions, a process controlled by steric constraints within the mRNA-binding pocket. eIF4A variants containing a Phe, Tyr, or His in position 163 facilitate π–π-stacking and are sensitive to rocaglates, while variants with Leu or Ser substitutions do not and are resistant (Table S2)^38^. To determine how other substitutions at position 163 affect π–π-stacking, we measured the shifts in thermal denaturation temperature of different eIF4A:RNA:rocaglate complexes.

We analyzed 18 natural and non-natural variants chosen based on the natural prevalence of particular aa substitutions, e.g., Phe, Tyr, Leu, or His, and/or their potential for establishing π–π-stacking interactions, e.g., Trp (Table S4). To evaluate the effect of mutations in position 199, we designed the variants to alternate between Ile, Val and Met.

**Figure 5:**
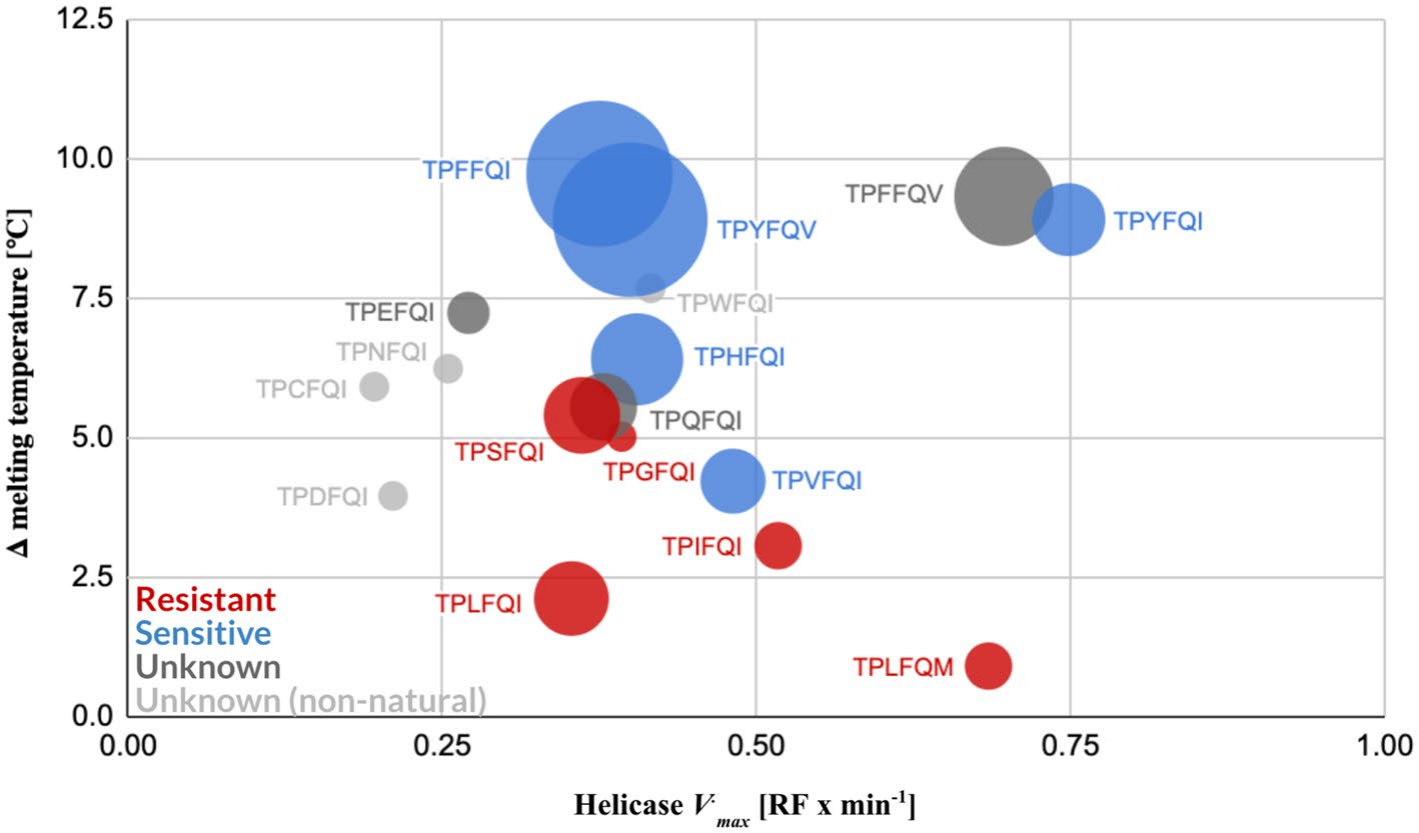
Shifts in thermal denaturation temperature of different eIF4A:RNA:silvestrol complexes are associated with eIF4A sensitivity to rocaglates. While relative eIF4A helicase activities of eIF4A mutant proteins expressing different rocaglate-binding aa patterns fall within a narrow range of *V*_*max*_ values and do not correlate with rocaglate sensitivity, comparative thermal shift analysis of the mutant proteins showed a clear association between sensitivity to rocaglates and higher thermal denaturation differentials between the eIF4A:RNA and eIF4A:RNA:silvestrol complexes. The increased stability of the rocaglate-sensitive mutants is determined by π–π-stacking interactions elicited by the corresponding aa residues at position 163. Data points represent mean values of three technical replicates. Standard errors for the helicase activities are indicated in Fig. 4A and standard errors for the *Δ* melting temperature are listed in Table S5. The size of the circles denotes prevalence of the aa pattern among the eIF4As included in our survey.

We determined thermal denaturation shifts, *Δ* melting temperature, for eIF4A:RNA complexes with three rocaglates: silvestrol, zotatifin, and CR-1-31-B. For silvestrol, larger *Δ* melting temperatures correlated with sensitivity to rocaglates (Fig. 5). Larger thermal denaturation shifts signify more stable eIF4A:RNA:rocaglate complexes requiring higher dissociation energies. The patterns exhibiting the largest temperature shifts were also the most well represented in our survey (Fig. 5). We observed equivalent thermal denaturation shift patterns for zotatifin and CR-1-31-B (Fig. S4).

All the measurements were of single or double aa variants on a human eIF4A1 protein scaffold. This analysis removed potential structural confounders but also preempted an evaluation of the substitutions in their naturally evolved structural context. For instance, the *Δ* melting temperature of purified *Aedes* *aegypti* eIF4A1 (TPHFQV) with silvestrol was 8.45 °C, higher than the 6.41 ℃ observed for the TPHFQI human eIF4A1 variant (Table S6). And an H163L mutant of the *Ae.* *aegypti* eIF4A exhibited a reduction by 2.35 ℃ in the Δ melting temperature to 6.1 ℃, mirroring the trend in melting temperature changes observed for the same aa 163 mutations (F163H and F163L) in the human eIF4A1 protein scaffold (Table S6). These results highlight the need to determine eIF4A sensitivity to rocaglates in their naturally evolved context to accurately quantify rocaglate sensitivity and RNA helicase activity.

### Sensitivity to rocaglates can be predicted through combined binding energy inference and thermal denaturation shift measurement analysis of eIF4A:RNA:rocaglate complexes

To assess whether rocaglate sensitivity could be predicted from in silico inference of the binding energies and intermolecular contact levels of eIF4A:RNA:rocaglate complexes, we performed a docking analysis of the eIF4A:RNA:rocaglate complexes of all 35 patterns with silvestrol, zotatifin, and CR-1-31-B in the human eIF4A1 background^38,52^. Both parameters were inversely correlated, and variants with the lowest binding energies and highest intermolecular contacts were the most abundant in our survey (Fig. S5). eIF4A variants that were shared with one or more other clades converged toward low binding energy/high intermolecular contact variants (Fig. S6).

**Figure 6:**
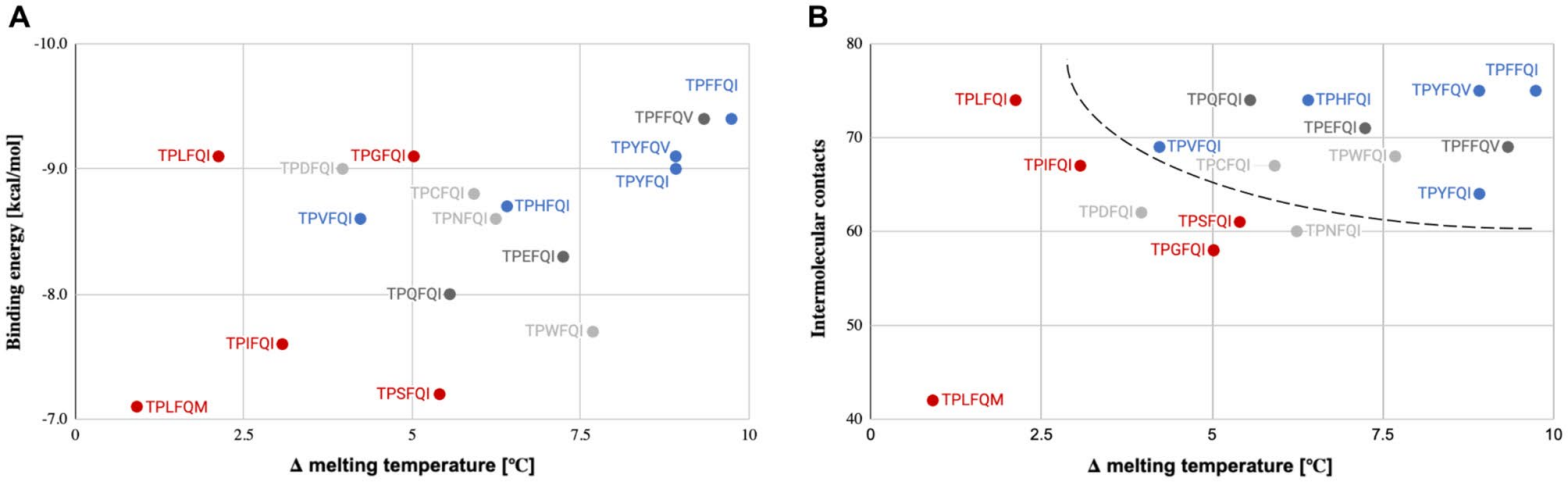
Combined binding energy or intermolecular contact inference with thermal denaturation shift measurement analysis of select eIF4A:RNA:silvestrol complexes reveals strong association with sensitivity to rocaglates. (**A**) Sensitivity to rocaglates (blue) was associated with high thermal denaturation shifts and low binding energies, while resistance (red) was associated primarily with low melting temperatures. Dark gray denotes natural eIF4A1 variants of untested resistance to rocaglates; light gray denotes non-natural eIF4A1 variants. (**B**) Sensitivity to rocaglates (blue) was associated with high thermal denaturation shifts and high intermolecular contacts, while resistance (red) was associated primarily with low melting temperatures. Dark gray denotes natural eIF4A variants of untested resistance to rocaglates; light gray denotes non-natural eIF4A variants; dashed line denotes approximate separation between rocaglate sensitive and resistant variants. Data points represent mean values of three technical replicates (*Δ* melting temperature) and single values from optimized docking analysis (intermolecular contacts). Standard errors for the *Δ* melting temperature are listed in Table S5.

Combined analysis of thermal denaturation shift measurements and inferred binding energies provided the strongest separation between sensitive and resistant variants of eIF4A, and intermolecular contacts and sensitivity to rocaglates were also associated (Fig. 6).

**Figure 7:**
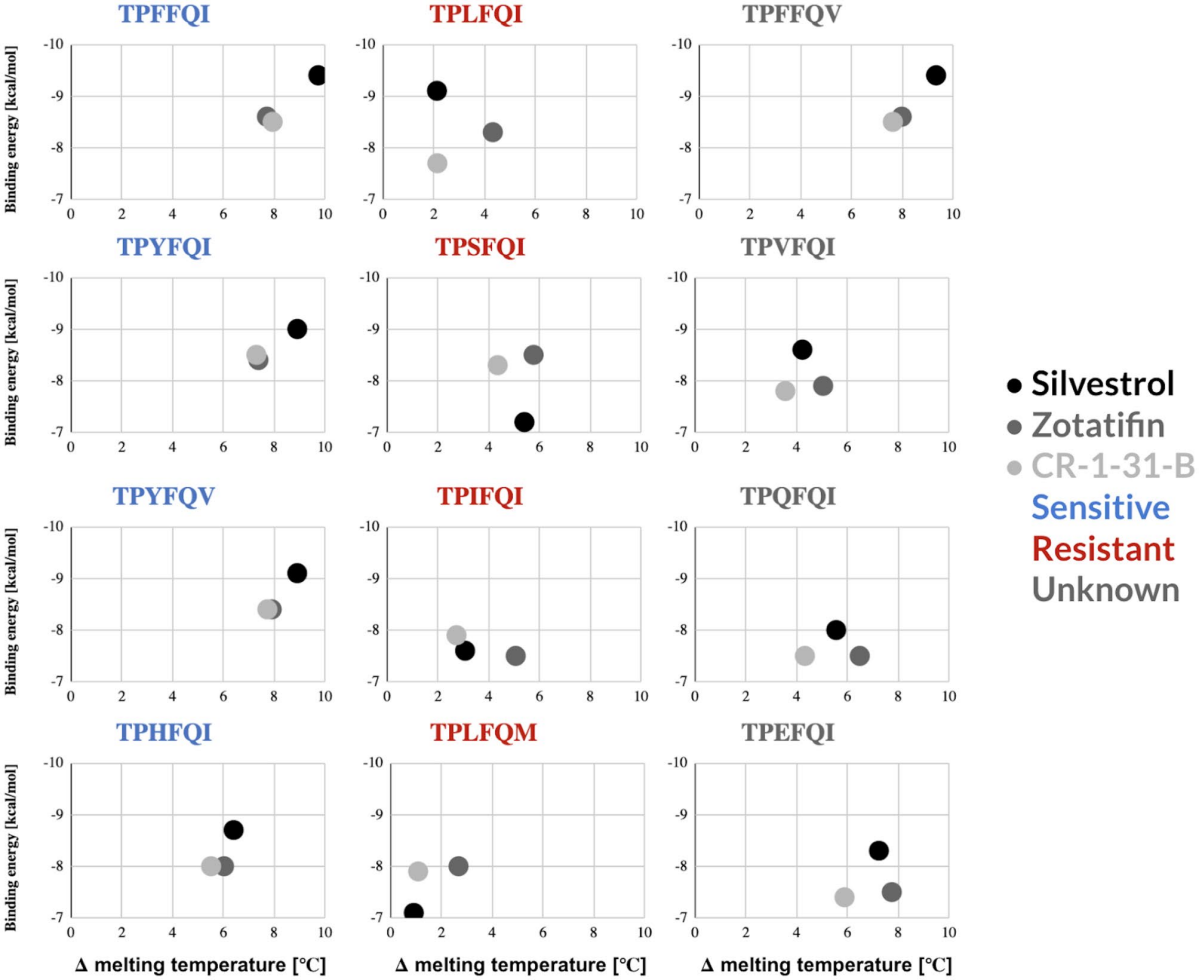
Side-by-side comparison of combined binding energy inferences and thermal denaturation shift measurements for silvestrol, zotatifin, CR-1-31-B. All eIF4A1 variants known to be sensitive to rocaglates (blue) exhibited remarkably similar patterns for all three rocaglates. By contrast, the four rocaglate-resistant variants analyzed here (red) showed widely disparate patterns. The four variants for which no experimental determination of sensitivity to rocaglates exists (dark grey), showed two distinct patterns—one that was analogous to that of the sensitive patterns analyzed here, and three that were analogous among them and different from all other sensitive or resistant patterns.

Analogous analyses with zotatifin and CR-1-31-B revealed a potentially predictive pattern of rocaglate sensitivity mirrored across four known sensitive variants—higher melting temperature shifts and binding energies for silvestrol and practically overlapping values for zotatifin and CR-1-31-B (Fig. 7). Another eIF4A variant, TPFFQV, which has not been evaluated in vitro yet, exhibited an almost identical pattern to the known rocaglate-sensitive variants (Fig. 7).

Structure-based computational modeling revealed that silvestrol may also interact with nearby Arg residues on the surface of eIF4A—Arg110, Arg282, and Arg311—via its 1,4-dioxane moiety (Fig. S7). These Arg residues belong to structurally distinct and conserved motifs of eIF4A: Arg110 is part of the PT**R**ELA sequence of motif Ia, Arg282 is part of the VIFCNT**R** sequence of motif IV, and Arg311 is part of the Qxx**R** motif. These three motifs fold into a conserved ‘Arg pocket’ adjacent to the RNA-binding pocket^42^. Arg311 also is involved in the formation of a critical salt bridge to the phosphate groups of RNA^38,53^.

Mutagenesis studies of the Arg pocket showed that single and triple substitutions of these residues to Ala result in a reduced ability of RNA to form a complex with eIF4A, resulting in varying degrees of clamping with silvestrol, RocA, and CR-1-31-B (Table S6). R282A mutation reduced the temperature shift in half, and R110A mutation resulted in no temperature shift. No addition of RNA to the assays resulted in consistent destabilization of the eIF4A association by about −3.32 ℃ (SD ± 0.53) (Table S6), indicating a reduced ability of the RNA to bind the R110A and the R282A mutants. The R311A and the triple mutants exhibited equivalent negative temperature shifts to those observed in all control assays without RNA addition, indicating an inability of the mutated eIF4A proteins to bind RNA (Fig. S8).

The rocaglate-resistant eIF4A variants—TPLFQI, TPIFQI, TPHFQI, and TPLFQM—showed no clear relative binding patterns for the three rocaglates we tested (Fig. 7). The extreme values of the experimentally and in silico determined values for the TPLFQM variant, including its optimal RNA helicase *V*_*max*_ point to this variant as evolutionarily favored in the context of *Aglaia* sp.

**Figure 8:**
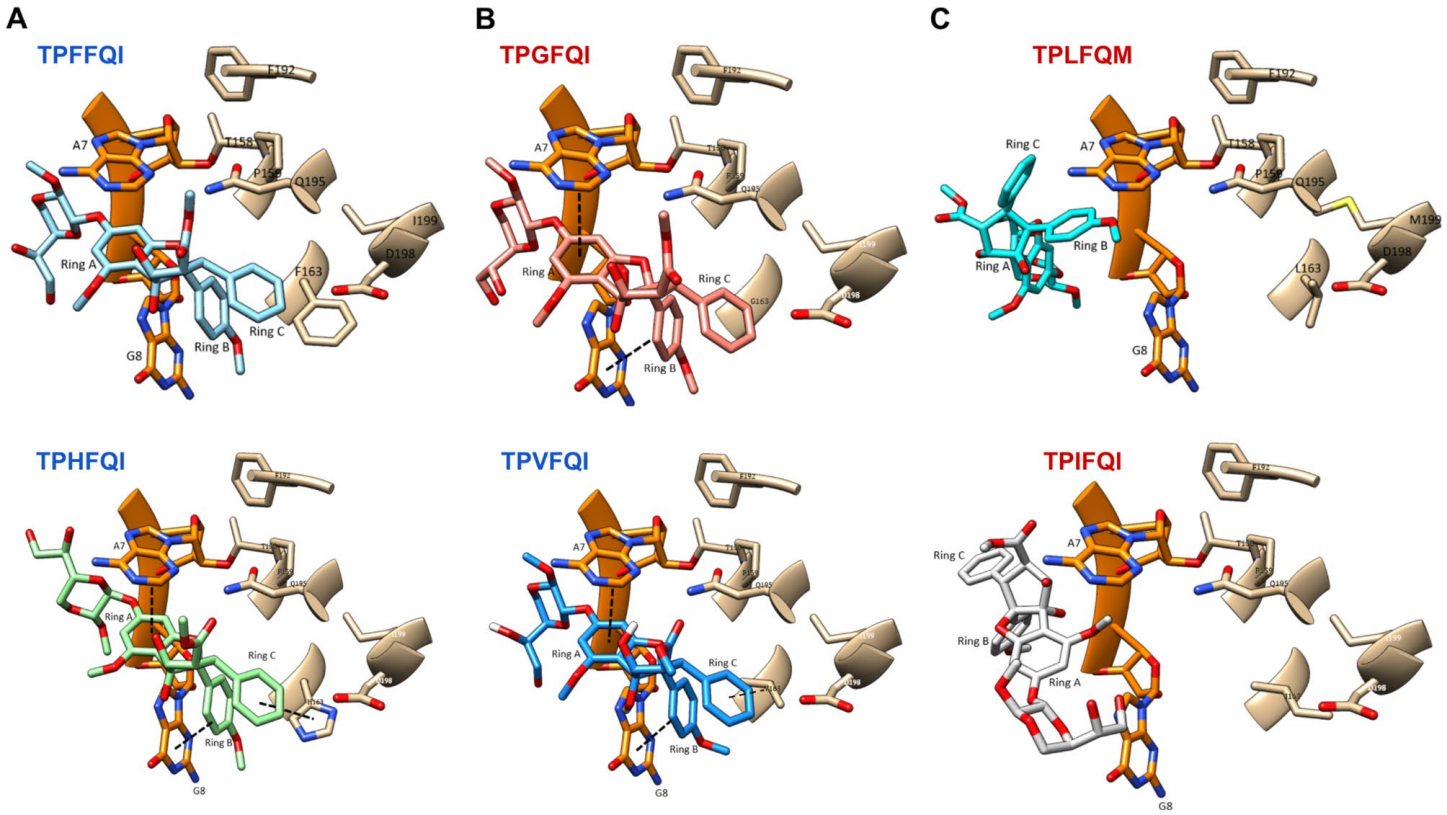
Molecular docking of silvestrol to select eIF4A variants. (**A**) Aromatic or aromatic-like aa residues at position 163 such as Phe or His, respectively, allow for the establishment of stable π–π-stacking interactions with rocaglates. (**B**) Substitutions with short chain aa residues such as Val allow for hydrophobic interactions with rocaglates that can to some extent compensate for the lack of π–π-stacking interactions. (**C**) Long aliphatic side chains such as Leu or Ile sterically preempt the formation of stable hydrophobic interactions, rendering the corresponding eIF4As resistant to rocaglate mediated clamping of the eIF4A:RNA complex.

The other natural eIF4A variants of unknown sensitivity we analyzed—TPVFQI, TPQFQI, and TPEFQI—exhibited analogous patterns among them, with lower binding energies for silvestrol than for zotatifin and CR-1-31-B, and consistently higher melting temperatures for zotatifin than for silvestrol or CR-1-31-B (Fig. 7). Based on the binding energies and intermolecular contacts inferred from the docking analysis, the Val residue at position 163 could mediate rocaglate-triggered clamping via hydrophobic interactions between phenyl ring C of the rocaglates and the aliphatic chain of Val, making this pattern sensitive to rocaglates (Fig. 8). The carbon backbone of Val is shorter than those of Leu and Ile, both of which have been shown to prevent the formation of π–π-stacking or hydrophobic interactions and did not allow for silvestrol to interact with eIF4A in our docking analysis (Fig. 8). The docking analysis predicted that a Val residue in position 163 might favor the establishment of stable hydrophobic interactions with rocaglates.

Docking analysis of the TPQFQI and TPEFQI mutants predicted the formation of stable hydrophobic interactions with rocaglates in analogous conformations to the π–π-stacking observed with Phe and Tyr or the aromatic-like basic aa His in position 163 (Fig. 8). Despite their differing chemical nature, Gln, an amine residue, and Glu, an acidic residue, exhibited similar binding energies and thermal denaturation shift patterns vis-á-vis silvestrol, zotatifin, and CR-1-31-B. This similarity, which implies the establishment of stable hydrophobic interactions, could be driven by the long carbon backbones of Gln and Glu.

### In vitro assays confirm predicted sensitivities to silvestrol

We performed in vitro silvestrol sensitivity assays with four species expressing eIF4As with previously untested aa patterns, four species with previously tested aa patterns but belonging to new genera, and one species belonging to a genus that had been previously characterized as rocaglate sensitive but had not been challenged with silvestrol (Table 1).

**Table 1:** List of organisms used for in vitro testing of silvestrol sensitivity (* motif inferred from 100% consensus among 32 fruit flies across five genera).

Species	Predicted sensitivity	Rocaglate-binding aa pattern	Measured sensitivity (S—sensitive; R—resistant)	Assay
*Schistosoma* *mansoni*	Sensitive	TPFFQI	S	Motility and egg production assays
*Anastrepha* suspensa* [AsE01]		TPYFQV	S	Cell proliferation assay
*Drosophila* *melanogaster* [S2]			S	Cell proliferation assay
*Plasmodium* *falciparum* [3D7]			S	Viability assay in erythrocytes
*Aedes* *aegypti* [Aag2]		TPHFQV	S	Cell proliferation assay
*Toxoplasma* *gondii*			S	Proliferation assay in MARC145 monkey kidney cells
*Trypanosoma* *brucei* *brucei* [Lister 427]		TPVFQI	S	Viability assay
*Leishmania* *amazonensis* [LV78]	Resistant	TPSFQI	R	β-lactamase assay in J774 macrophage cells
*Caenorhabditis* *elegans*		TPGFQV	R	Developmental and lifespan assays

We selected two pathogens—*Toxoplasma* *gondii* and *Trypanosoma* *brucei* *brucei*—the pathogen vector *Aedes* *aegypti,* and the non-pathogenic nematode *Caenorhabditis* *elegans* to test whether our predictions on eIF4A sensitivity could be confirmed. *Ae.* *aegypti*, *T.* *gondii* and *T.* *brucei* *brucei* were predicted to be sensitive to silvestrol based on their aa substitutions: F163H (*Ae.* *aegypti*, *T.* *gondii*) and F163V (*T.* *brucei* *brucei*), and *C.* *elegans* was predicted to be resistant to silvestrol (F163G). Indeed, by using model-specific viability, developmental or lifespan assays, we were able to confirm all three predictions (Figs. S9 and S10).

We complemented the series by testing another important pathogen, *Schistosoma* *mansoni*, and the non-pathogenic fruit flies *Anastrepha* *suspensa* and *Drosophila* *melanogaster*. The eIF4A sequences of *S.* *mansoni* and *D.* *melanogaster* contained aa patterns TPFFQI and TPYFQV, respectively, which had been previously shown to be sensitive to rocaglates. The eIF4A sequence of *A.* *suspensa* was not available at the time of this writing, but the eIF4A mRNA sequence of the closely related *A.* *fraterculus* was available (TPYFQV)^54^ and, given the 100% consensus of this motif across five genera and 32 species of fruit flies (Table S2), we assumed the presence of the same motif in *A.* *suspensa*. With these assays we were able to confirm the applicability of sensitivity results from one species to unrelated genera. In addition to *T.* *brucei*, we also tested two other protozoan pathogens—*Leishmania* *amazonensis* and *Plasmodium* *falciparum*—to confirm that aa patterns associated with silvestrol sensitivity in one species would confer sensitivity to different species within the same genus containing the same aa pattern. We also observed that two protozoans within the same family—*T.* *brucei* and *L.* *amazonensis*—exhibited rocaglate sensitivity and resistance, respectively, in concordance with their corresponding eIF4A aa patterns (Figs. S10 and S11; Table 1).

All assays confirmed the predictions, expanding the number of potential pathogens that can now be targeted with rocaglates from the 50% we initially estimated based on known sensitivity reports to 60% (Table S2). The proportion of potentially resistant pathogens rose from 13 to 19% (Table S2).

The combined binding energy and thermal denaturation shift analysis showed analogous patterns for three variants of unknown sensitivity to rocaglates: TPVFQI, TPEFQI, and TPQFQI (Fig. 7). The in vitro results revealing the sensitivity of TPVFQI to silvestrol suggests that organisms containing the analogous aa combination TPEFQI and TPQFQI could also be sensitive to rocaglates, further expanding the list of pathogens and other harmful organisms targetable with rocaglates (Table S2).

## Discussion

### Enlisting rocaglates to fight eukaryotic pathogens

The six aa residues that determine eIF4A sensitivity to rocaglates provide a tool to predict sensitivity. Our mutant analysis provides the first in vitro confirmation for three predictions: TPVFQI, and TPHFQV (sensitive), and TPGFQV (resistant).

TPVFQI, present in *Trypanosoma* sp., opens the possibility of targeting *T.* *brucei* (sleeping sickness), and *T.* *cruzi* (Chagas disease). TPHFQV is present in agricultural pathogens including *Cystoisospora* *suis* and *Marssonina* *coronariae*, and in high-impact disease vectors *Aedes* *aegypti* and *Ae.* *albopictus*. TPGFQV, which with the exception of *Caenorhabditis* *elegans* is only found in protists, rules out the use of rocaglates or its derivatives for pathogens including *Phytophthora* sp., *Aphanomyces* sp., and *Saprolegnia* sp. The analogous resistant variant TPGFQI has so far only been identified in the *Aglaia* sp. fungal parasite *Ophiocordyceps* sp..

Sensitivity could be predicted by in silico modeling of binding energies and intermolecular contact levels of the ternary eIF4A:RNA:rocaglate complex and by in vitro analysis of the melting temperatures of the eIF4A:RNA complexes. Our analysis shows the potential for enlisting rocaglates to fight pathogens, but several important questions remain.

First, the diversity of eIF4A variants we report might represent only a fraction of their true natural diversity, but it provides insights into potential evolutionary constraints determining resistance to rocaglates that might be critical to preempting the emergence of further resistance.

Secondly, our analysis has shown the effect of single aa substitutions on intermolecular dynamics in a fixed human background, but how those variants perform in their natural protein scaffolds will ultimately have to be determined in their native scaffolds.

And thirdly, the interaction of different rocaglates with eIF4A varies in subtle ways that are not yet completely understood. For instance, the TPIFQI variant elicits analogous interactions with silvestrol and CR-1-31-B that are distinct from those with zotatifin and can thus not be solely explained by the presence of silvestrol’s 1,4-dioxane moiety. Understanding these natural interactions will further inform the design of rocaglates of improved efficacy and specificity.

### Does natural resistance to rocaglates provide a fitness advantage?

Mapping the eIF4A variants onto the eukaryotic tree of life—a widely accepted proxy for an evolutionary timeline—revealed a random distribution of resistance variants. Given that known rocaglate biosynthesis is limited to one plant genus, *Aglaia*, which would have emerged at a much later stage than most of the resistant organisms we identified, and that a functional role of rocaglates in *Aglaia* sp. is not known, two scenarios for the emergence of rocaglate resistance can be explored.

First, resistance could emerge following exposure to rocaglate biosynthesized by *Aglaia*. The recent description of the *Aglaia* sp. parasitic fungus *Ophiocordyceps* sp. BRM1 supports such a scenario. However, many other rocaglate-resistant organisms have non-overlapping distributions with that of *Aglaia*, or global distributions, making it unlikely that rocaglates provided an evolutionary pressure.

Alternatively, rocaglate resistance could be a neutral byproduct of natural eIF4A variation. This would be supported by the fact that the relative *V*_*max*_ of the different natural eIF4A variants we analyzed was remarkably constant, and that *Aglaia* sp. and *Ophiocordyceps* sp. BRM1 are the only organisms in which both eIF4A isoforms are resistant to rocaglates, protecting them from self-poisoning and poisoning by the host, respectively.

None of these scenarios can be ruled out because aa substitution tolerance could be driving the random appearance of ‘resistant’ variants, and physical proximity and exposure to rocaglates could be triggering the retaining of ‘resistant’ variants simultaneously.

### Thoughts on the emergence of de novo resistance to rocaglates in pathogens

We have identified potential targets for rocaglates based on the eIF4A sequences of pathogens of interest for human, animal and plant health. The data also highlight the potential for de novo development of rocaglate resistance and the need for carefully managing any anti-pathogen applications of rocaglates.

The deployment of antimicrobials has taught us that nature will always adapt to new challenges through natural evolution and survival of the fittest^55^. This has resulted in the emergence of resistance to antimicrobials, whether through lateral transfer of genes from other microorganisms, de novo mutations, or repurposing of existing mechanisms for detoxification.

The emergence of resistance to pesticides in more complex organisms such as fungi and insects has been tied to factors including the number of point mutations needed for resistance, the pre-existence of resistance alleles in a population, and the fitness of the mutated resistant variants in the absence of the corresponding selection pressure^56,57^.eIF4A resistance to rocaglates could be an accidental trait arising from the aa substitution tolerability at position 163. Single point mutations leading to aa changes at this position can turn a rocaglate-sensitive organism into a resistant one. Rocaglate-driven clamping is mainly determined by the aa residue at position 163, however, this happens within the constraints of four other residues having to remain unchanged—158, 159, 192, and 195—and one being tolerant to minimal substitution—199. Clamping also requires the presence of two adjacent purine RNA bases to stabilize the eIF4A1:RNA complex, which constrains the tolerability for substitutions at the relevant RNA interacting aa residues of eIF4A^40^. The combination of these factors makes the emergence of resistance a more complex albeit overall still low barrier evolutionary event.

A priori, the natural diversity of rocaglate-resistant eIF4A alleles would seem to pose a major barrier to the implementation of rocaglate-based anti-pathogen strategies. However, this extensive catalog of information about sequence, structural and physicochemical factors that characterize ‘resistant’ alleles could be used to inform the development of novel compounds, the deployment of carefully managed control programs, and the monitoring of the emergence of potential de novo resistance in managed populations.

‘Fitness rescue’ in species containing a resistant and a sensitive isoform has so far only been shown in cases where eIF4A1 is the resistant isoform but not when it is eIF4A2 that contains the resistant allele. This is the case for most of the pathogens we have analyzed so far. And while we have no way of knowing how fast resistant mutations have been acquired over evolutionary timescales, our analysis has revealed that, at least in theory, resistant mutations could arise quickly through point mutation events at the codon wobble position as exemplified by the switch from Phe to Leu.

All natural variants of eIF4A exhibit relative *V*_*max*_ within a narrow range that is most likely essential for the fitness of the organism. This applies to both resistant and sensitive variants of eIF4A, suggesting that fitness would not be a deciding factor for the permanent establishment of a resistant allele.

Given the minimal number of aa substitutions needed for resistance, the pre-existence of resistance alleles in the population, and the neutral fitness advantage of resistant variants in the absence of rocaglates, the opportunity for the application of rocaglates as anti-pathogens is complex. While the listed factors seem to compromise the potential of harnessing rocaglates for managing pathogens, this knowledge, which was often lacking prior to the implementation of other anti-pathogen compounds, could provide the basis for more robust and sound strategies. Measures such as punctual, high concentration deployments of rocaglates accompanied by comprehensive monitoring programs could be one approach to minimizing the emergence of resistance. Taking advantage of the favorable therapeutic windows of rocaglates in humans and animals, compared to the sensitivity in fungi and protists, could be pivotal in developing safe interventions against select rocaglate-sensitive parasites. Harnessing the comprehensive catalog of natural eIF4A variants uncovered in our study could further help advance the search for novel synthetic rocaglates or other small molecule inhibitors of enhanced specificity and efficacy.

## Methods

### eIF4A sequence analysis

Sequence analysis was performed using the full eIF4A protein sequences retrieved from Genbank and cross-validating them on UniProt to determine specific isoforms. Only sequences corresponding to the eIF4A1 and eIF4A2 isoforms were used for the analysis. Next, we extracted the six amino acid motifs associated with rocaglate binding for each of the protein sequences (human positions 158, 159, 163, 192, 195, 199)^38,39^. Variant motif distributions were determined within each eukaryotic grouping and mapped onto the latest version of the eukaryotic tree of life^51^. Sequence logos illustrating amino acid tolerances for the six amino acids analyzed were rendered using Seq2Logo-2.0^50^.

### eIF4A variant cloning, overexpression and purification

Select eIF4A variants with single and double substitutions at positions 163 and/or 199 were generated using PCR-based site-directed mutagenesis of a plasmid encoding human eIF4A1 (pET-28a( +)_eIF4A1(19-406) containing an *N*-terminal His-Tag and a thrombin cleavage site; variant-specific primers listed in Table S4). Double mutants were generated by first generating the mutations at position 163 followed by the mutations at position 199. Ligation products were transformed into *E.* *coli* DH5α cells and the plasmids were sequenced to confirm the corresponding substitutions. Following sequence confirmation, competent *E.* *coli* BL21 (DE3) cells were transformed with the plasmids and grown in lysogeny broth medium at 37 °C to OD_600_ ~ 0.5. After the addition of 0.5 mM IPTG, the cells were grown at 15 °C for 16 h. The collected cells were lysed by sonication in a 20 mM HEPES–KOH buffer (pH 7.5, 300 mM KCl, 20 mM imidazole, 5 mM β-mercaptoethanol, 0.1 mM EDTA, 10% (v/v) glycerol) with 1× cOmplete™, Mini, EDTA-free Protease-Inhibitor-Cocktail (Roche). The lysate was fractionated on a HisTrap™ HP 1 mL column (GE Healthcare) using a linear gradient from sonication buffer to elution buffer (sonication buffer with 250 mM imidazole). The peaked fractions were collected, buffer-exchanged to a 20 mM HEPES–KOH buffer (pH 7.5, 300 mM KCl, 5 mM MgCl_2_, 0.1 mM EDTA, 1 mM DTT and 10% (v/v) glycerol), flash-frozen in liquid nitrogen and stored at –80 °C.

### Helicase assay

Helicase activities of the eIF4A variants were determined using a fluorescence-based assay. The capacity to unwind dsRNA substrates was measured using two labelled RNA substrates: a 10mer modified with Cyanine 3 (10mer-Cy3; 5′-[CY3]GCUUUCCGGU-3′), and a 16mer modified with Black Hole Quencher 2 (16mer-BHQ2; 5′-ACUAGCACCGGAAAGC[BHQ2]-3′). An unlabeled competitor (10mer-competitor; 5′-GCUUUCCGGU-3′) was used to capture released quencher RNA. A single-stranded Cy3 RNA substrate (ssRNA) was used to determine the maximum fluorescence signal of the reaction. Equimolar amounts of 10mer-Cy3 and 16mer-BHQ2 were annealed at 80 °C for 5 min and incubated at room temperature for 1 h followed by incubation on ice for 10 min in a 25 mM HEPES (pH 7.4 (KOH) in ddH_2_O). Competitor RNA was added in 1:10 (v/v) excess to the labelled RNA substrates and the reaction was again incubated on ice for 10 min prior to adding it to the helicase reaction mix. eIF4A (25 µM final concentration) was added to the reaction and fluorescence was measured using a Safire 2 microplate reader (Tecan).

### Thermal shift assay

Thermal shift assays were performed by incubating 5 μM of recombinant human eIF4AI (19-406) with 50 μM of a polypurine RNA (AG)_5_ (Biomers, Ulm, Germany), 1 mM AMP-PNP (Roche, Basel, Switzerland), 100 μM of rocaglate (silvestrol, RocA, zotatifin, or CR-1-31-B) and 75 μM SYPRO Orange (S6650, Invitrogen, Carlsbad, CA, USA) in a 20 mM HEPES–KOH buffer (pH 7.5, 100 mM KCl, 5 mM MgCl_2_, 1 mM DTT, 0.1 mM EDTA, and 10% (v/v) glycerol) for 10 min at RT. The melt curves were measured between 10 and 95 ℃ at a 1.6 ℃/min ramp rate using the QuantStudio3™ Real-Time PCR system (Applied Biosystems, Waltham, MA, USA) in a MicroAmp™ Fast Optical 96-well plate (Applied Biosystems, Waltham, MA, USA).

### Docking analysis

Molecular docking was performed using AutoDock, v4.2^58^. The proteins were processed by adding all hydrogen atoms and merging non-polar hydrogen atoms using AutoDock Tools 1.5.7. Charges were assigned using the Gasteiger method with fixed torsions for the ligand. We set a 60 × 60 × 60, 3.75 Å grid box around the active sites with x, y and z-dimensions of 46.355, 9.919, 47.473, respectively. The rigid grid box was set using AutoGrid 4, followed by AutoDock with the Lamarckian genetic algorithm to obtain the best docking poses^59^. Dockings were performed in duplicate and the average binding energy reported. Select poses representing optimal binding affinities were visualized using UCSF Chimera (University of California).

### Cell-based in vitro studies

#### *Aedes aegypti*, *Anastrepha suspensa*, and *Drosophila melanogaster*

Cell proliferation assays were performed with insect cell lines (Aag2 [*A.* *aegypti*]^60^, AsE01 [*A.* *suspensa*]^61^, and S2 [*D.* *melanogaster*] (ThermoFisher)) using the WST-1 assay (Sigma-Aldrich). Cells (Aag2: 1.2 × 10^5^ cells/100 μL, AsE01: 1 × 10^5^ cells/100 μL, S2: 6 × 10^4^ cells/100 μL) were incubated in the presence of silvestrol (0 nM to 1.6 µM). Following a 24 h incubation with silvestrol, we added WST-1 reagent as specified by the manufacturer and waited an additional three hours before determining cell mortality by measuring absorbance at 440 nm (reference wave length: 600 nm). CC_50_ values were determined for each set of biological replicates measured (GraphPad Prism V9).

### Whole organism-based in vitro studies

#### Toxoplasma gondii

The effect of silvestrol treatment on *Toxoplasma* *gondii* replication in MARC-145 cells [Elabscience] was determined at 48 h post infection (h p. i.). Cell viability was controlled after 48 h of treatment with up to 100 nM silvestrol via XTT assays (solvent: DMSO (1:500); positive control: Triton X-100 treatment (1:200); negative control: plain medium). At 48 h p. i., the number of *T.* *gondii* tachyzoites released from infected host cells into the cell supernatant was determined. Assays were performed in triplicate.

#### Trypanosoma brucei brucei

Viability assays of *T.* *brucei* *brucei* (non-recombinant 427 strain) were performed for 48 h using HMI-9 medium (modified DMEM (IMDM; Cell Gro); 10% FBS; 10%, Serum plus (SAFC); 0.05 mM Bathocuproinesulfonate; 1.5 mM l-cysteine; 1 mM hypoxanthine; 0.2 mM β-mercaptoethanol; 0.16 mM thymidine; 1 mM pyruvate). After a 48 h incubation, cells were labeled with Alamar blue and fluorescence measured at 530 nm and 590 nm. All assays were performed in triplicate.

#### Caenorhabditis elegans

Developmental and lifespan assays were conducted with N2 wild type *C.* *elegans* worms reared on NGM agarose plates infused with silvestrol and seeded with 30 µl OP50 *E.* *coli*/LB medium. For the developmental assays, ten worms were allowed to lay eggs for 2 h (synchronization), single eggs were isolated on separate experimental plates and incubated at 20 ℃, and the first egg laying event after 59 h was determined. Number of progeny was determined by counting the total number of progeny from synchronized, isolated animals^62^. For the lifespan assays, 40 worms were allowed to lay eggs for 2 h (synchronization), 15 eggs per plate were transferred to experimental plates and incubated at 20 ℃, and after 3 days, mothers were transferred to a fresh plate every other day until day eight of adulthood to avoid overgrowth by the progeny. Live worms were counted daily until all of them died. Unnatural deaths were removed from the analysis. All assays were done at least in triplicate.

#### Schistosoma mansoni

Adult worm couples were cultured in M199 medium (Sigma-Aldrich, Germany) supplemented with 10% newborn calf serum, 1% 1 M HEPES and 1% ABAM solution (10,000 units/ml penicillin, 10 mg/ml streptomycin and 25 mg/ml amphotericin B) at 37 °C in a 5% CO_2_ atmosphere. Activity of silvestrol (100 and 200 nM) against the worms was evaluated for seven days in vitro. Medium and silvestrol were refreshed daily and worm motility as well as the number of laid eggs assessed after three and seven days using an inverted microscope (Labovert, Germany). Worm motility was scored as recommended by WHO-TDR^63^, where a score of ‘3’ indicates normal motility, ‘2’ reduced motility, ‘1’ minimal and sporadic movements, and ‘0’ represents dead worms (no movement within 30 s). Worms were obtained from infected hamsters as described elsewhere^64^.

All animal experiments with Syrian hamsters (*Mesocricetus* *auratus*) were conducted in accordance with the European Convention for the Protection of Vertebrate Animals used for Experimental and Other Scientific Purposes (ETS No 123; revised Appendix A), were approved by the Regional Council (Regierungspräsidium) Giessen, Germany (V54-19 c 20/15 h 02 GI 18/10 Nr. A 14/2017), and are reported in compliance with the ARRIVE guidelines.

#### Leishmania amazonensis

Promastigotes of a *L.* *amazonensis* strain expressing β-lactamase^65^ were cultured in immortalized J774 macrophage cells [ATCC] grown in RPMI supplemented with 10% of FBS and 1% PSG. Briefly, duplicate assays with silvestrol and triplicate controls were performed with plated macrophages infected with stationary phase *L.* *amazonensis* (25 parasites/macrophage) and incubated overnight at 32 °C and 5% CO_2_. Serial dilutions of silvestrol were added and incubated for 96 h at 32 °C and 5% CO_2_. Viability assays were conducted using CENTA™ β-Lactamase Substrate (EMD Chemicals) and Nonidet P-40 (Igepal CA 360, Fluka), and absorbance was measured at 405 nm.

#### Plasmodium falciparum

Fluorescence-based viability assays were conducted for 96 h with erythrocytic asexual cultures (5% hematocrit) of *P.* *falciparum* strain 3D7 (0.25% ringstage parasitemia; synchronous) in RPMI medium (RPMI 1640; 25 mM HEPES; 10 ug/ml gentamycin; 0.5 mM hypoxanthine; pH 6.75; 25 mM sodium bicarbonate; 0.5% Albumax II; 1% O_2_, 5% CO_2_: 94% N_2_)^66^. Viability was determined by quantifying fluorescence following staining of *P.* *falciparum* cells with SYBR Green I (Molecular Probes).

## Supplementary Information

Supplementary Figures.

Supplementary Table S1.

Supplementary Table S2.

Supplementary Table S3.

Supplementary Table S4.

Supplementary Table S5.

Supplementary Table S6.

Supplementary Table S7.

## Data Availability

All the sequence data analyzed in this study were retrieved from Genbank. The two new sequences corresponding to *Aglaia* *stellatopilosa* and *Aglaia* *glabriflora* have been deposited in GenBank (Accession numbers ON844099 [Aglaia stellatopilosa voucher SBC6708 eukaryotic initiation factor 4a (eiF4A) mRNA, partial cds] and ON844100 [Aglaia glabriflora voucher SBC0002 eukaryotic initiation factor 4a isoform 1 (eIF4A) mRNA, partial cds], respectively). All other data presented in this study are reported in full in the Supplementary Tables.
