# Supplementary material for: Broad anti-pathogen potential of DEAD box RNA helicase eIF4A-targeting rocaglates
Source: Sci Rep. 2023 Jun 8;13:9297. doi: 10.1038/s41598-023-35765-6 (PMC10250355; doi:10.1038/s41598-023-35765-6)
Supplement: Supplementary file 1 — Supplementary Figures. [file 41598_2023_35765_MOESM1_ESM.pdf]

Supplemental materials

Table S1 – Summary of pathogens that have been previously tested for their sensitivity to a range of natural and synthetic rocaglates

Table S2 – List of rocaglate-associated aa motifs extracted from a global survey of eIF4A protein sequences

Table S3 – List of organisms containing eIF4A isoforms with differing rocaglate-associated aa motifs

Table S4 – List of eIF4A mutants generated for this study

Table S5 – Mutant temperature shift analysis

Table S6 – Native *Aedes aegypti* eIF4A temperature shift analysis

Tabel S7 – Arg pocket analysis

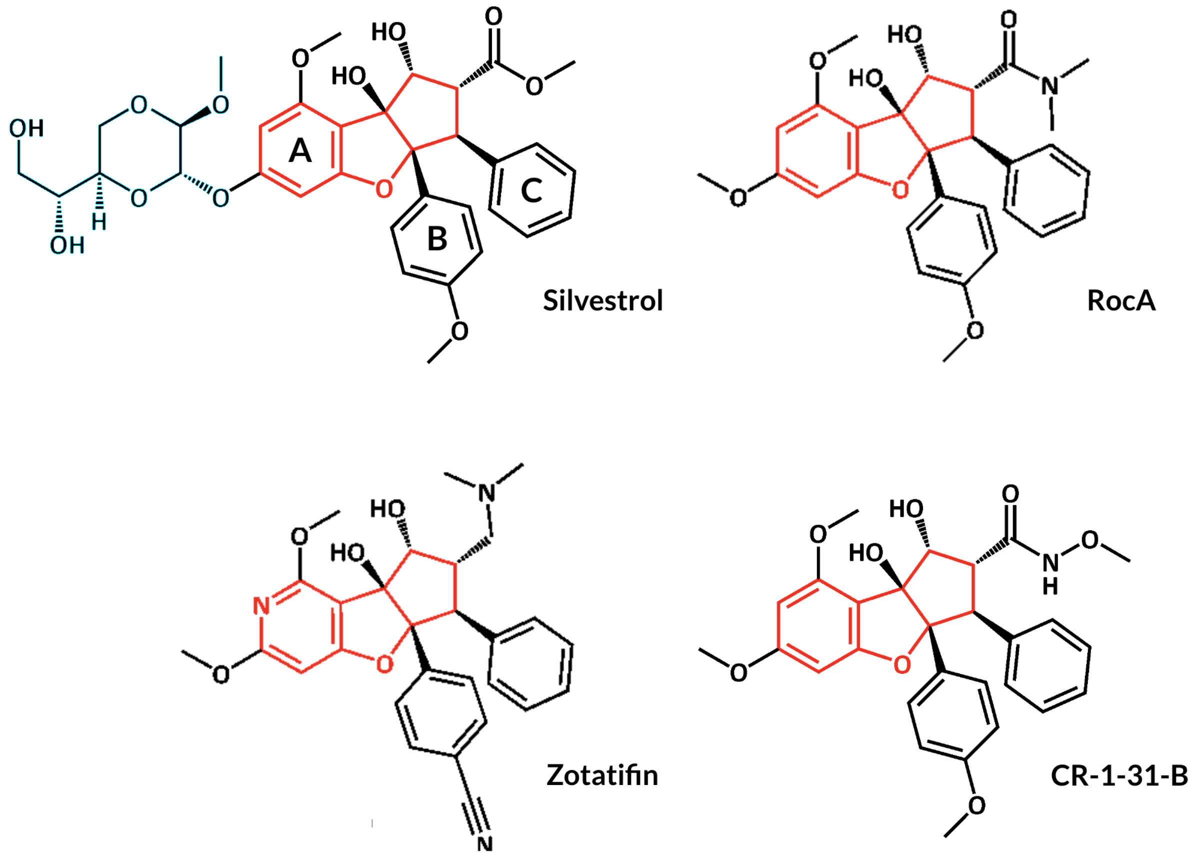

Figure S1: Chemical structures of the four rocaglates tested in this study. All rocaglates are characterized by a cyclopenta[*b*]benzofurane skeleton, indicated in red, and three benzyl rings, A, B, and C. Silvestrol, the archetypal natural rocaglate, contains a unique 1,4-dioxane moiety, indicated in blue, that increases the possibility of interactions with aa residues beyond those in the RNA-binding pocket. RocA, the first natural rocaglate to be purified and structurally characterized, exhibits the core structure of natural rocaglates. Synthetic rocaglates zotatifin and CR-1-31-B exhibit nitrile and imido groups, respectively, that modulate the binding characteristics of the molecules to the eIF4A:RNA complex.

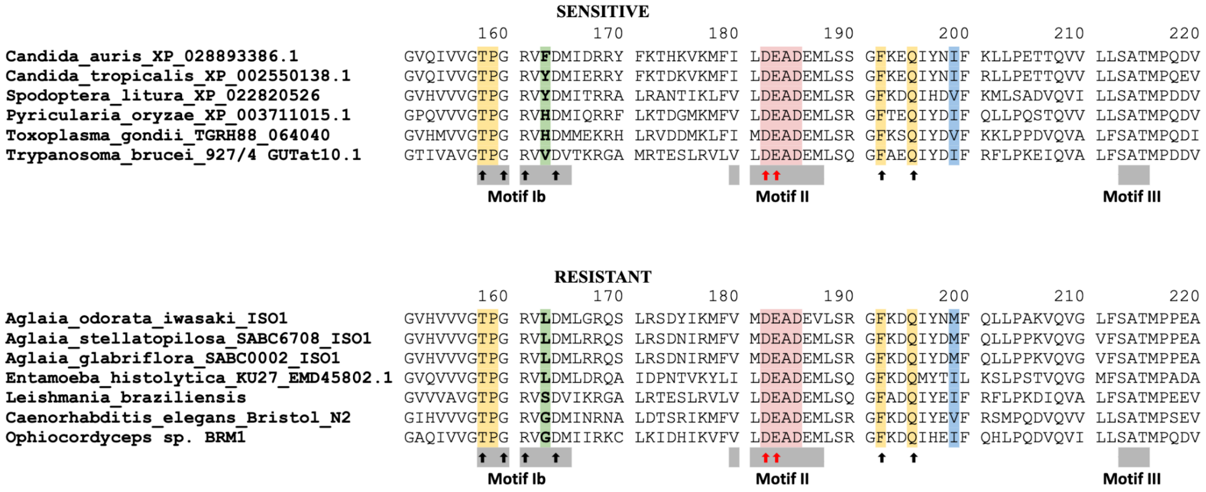

Figure S2: Partial aa sequence alignment of eIF4A proteins encoded by rocaglate sensitive and resistant organisms. The alignment shows the RNA-binding pocket of eIF4A, which comprises the conserved amino acid sequence Asp-Glu-Ala-Asp (D-E-A-D) characteristic of ATP dependent DEAD-box RNA helicases, motifs Ib, II, and III, which are characteristic of eIF4A proteins, and six aa residues critical to rocaglate binding. The aa residues at positions 158, 160, 161, 164, 192, and 195 (black arrows) are involved in the protein's interaction with RNA, and residues 182 and 183 (red arrows), located within the DEAD-box (pink), are involved in the interaction with ATP. Position 163 is the primary determinant of sensitivity to rocaglates (green), followed by aa residue 199 (blue) and four conserved aa residues at positions 158, 159, 192, and 195 (yellow). The sequences shown are representative of eIF4A proteins with aa patterns that have been shown to confer rocaglate sensitivity or resistance, including two novel sequences of the rocaglate-producing plants *Aglaia stellatopilosa* and *A. glabriflora*, both reported in this study (Accession numbers ON844099 and ON844100, respectively).

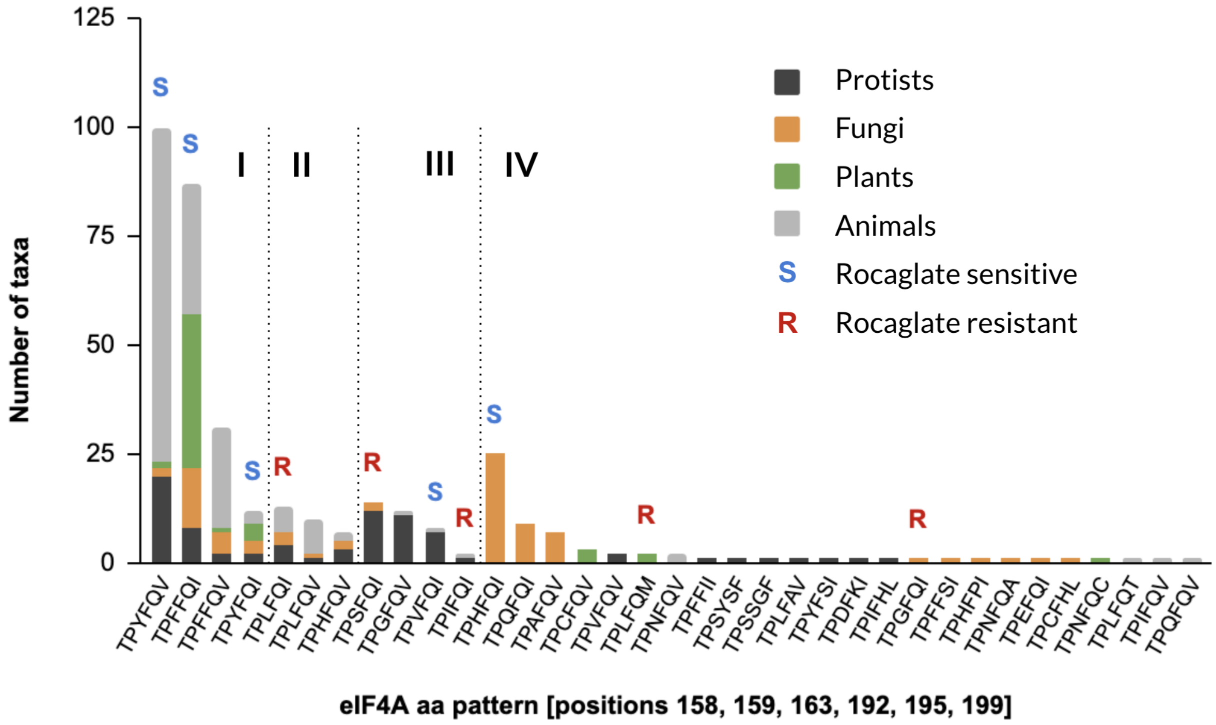

Figure S3: Representation of patterns of aa critical for rocaglate-binding in known eIF4A proteins across the four main groups of eukaryotes. A comprehensive analysis of known eIF4A proteins revealed 35 patterns of aa at positions 158, 159, 163, 192, 195, and 199 (human numbering). Four primary aa patterns were present in all four groups of eukaryotes, representing 63% of all eIF4As (I). Another three patterns were present in three groups (II) and four were present in two groups (III). The largest proportion of patterns, 71%, was only present in one group of eukaryotes and in most cases with only one representative species (IV). Known natural resistance is restricted to only four patterns, including two patterns—TPLFQM and TPGFQI—unique to members of the plant genus *Aglaia sp.*, so far, the only organism known to biosynthesize rocaglates, and its fungal parasite *Ophiocordyceps* sp. BRM1, respectively.

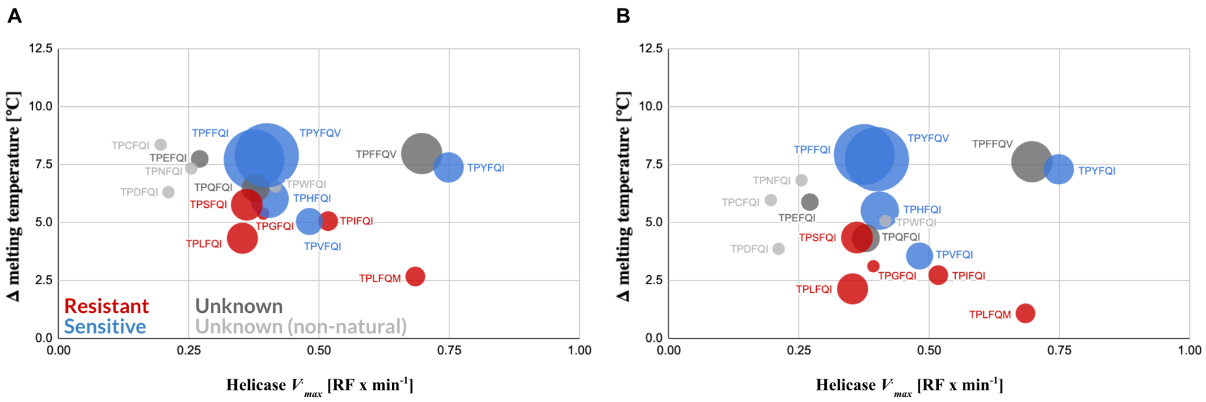

Figure S4: Shifts in thermal denaturation temperature of different eIF4A:RNA:zotatifin and eIF4A:RNA:CR-1-31-B complexes and their association with eIF4A sensitivity to rocaglates. The complexes established between eIF4A:RNA with two artificial rocaglates, zotatifin (**A**) and CR-1-31-B (**B**), exhibited thermal shift patterns showing a clear association between sensitivity to rocaglates and higher thermal denaturation differentials similar to those exhibited by the equivalent eIF4A:RNA:silvestrol complexes. The complexes also showed similar helicase V_{max} ranges to those determined for the equivalent eIF4A:RNA:silvestrol complexes (**Figure 4**). Data points represent mean values of three technical replicates. Standard errors for the helicase activities are indicated in Fig. 4A and standard errors for the ***Δ*** melting temperature are listed in **Table S5**. The size of the circles denotes prevalence of the aa pattern among the eIF4As included in our survey.

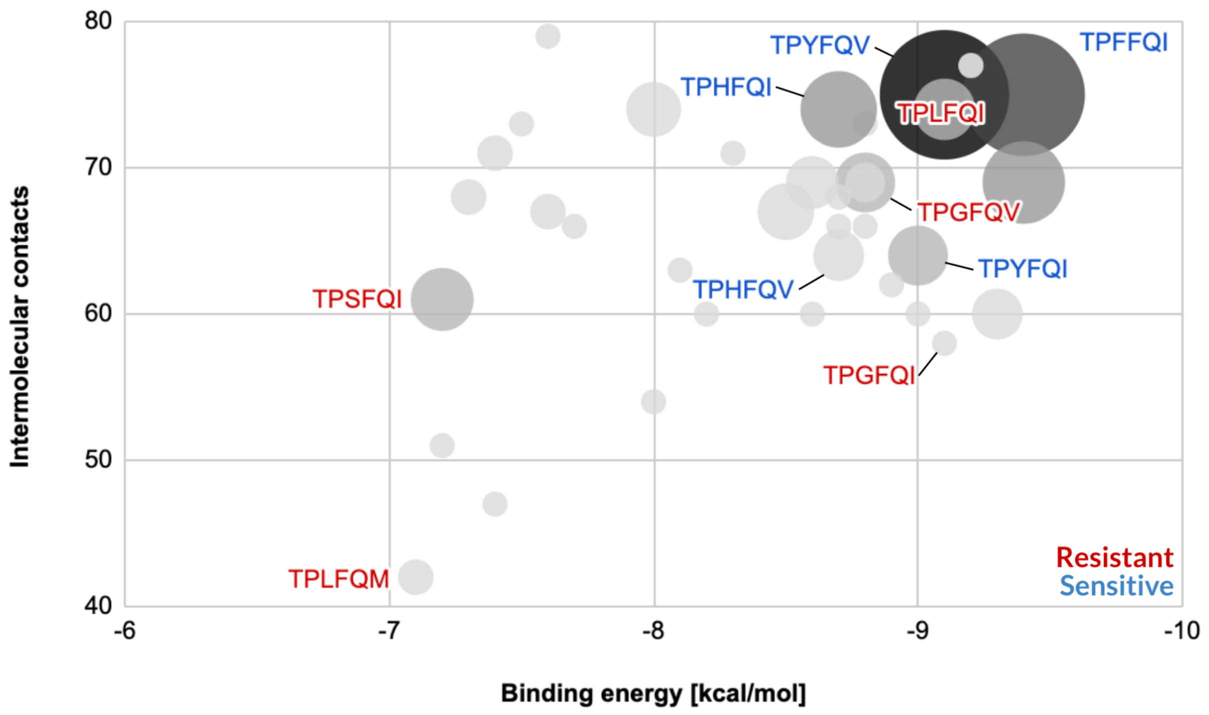

Figure S5: The binding energies and intermolecular contact levels of natural eIF4A:RNA:silvestrol complexes are highly correlated and exhibit an overall skew toward low binding energies and high intermolecular contacts. While rocaglate-sensitive eIF4A variants (shown in blue) exhibited the lowest binding energies and the highest intermolecular contacts, resistance to rocaglates was not preferentially associated with either of these parameters (shown in red).

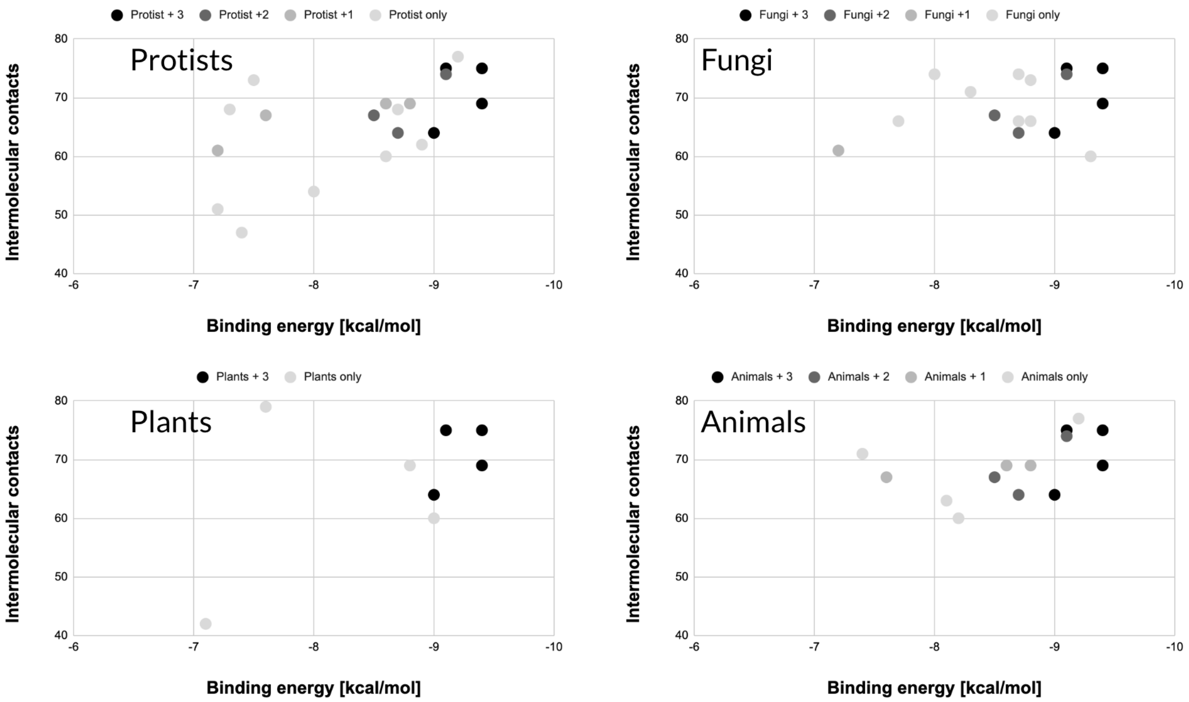

Figure S6: Convergence of eIF4A variants toward low binding energy/high intermolecular contact variants relative to silvestrol. In all four groups of eukaryotes analyzed—protists, fungi, plants, and animals—the inferred binding energy and intermolecular contacts converged toward variants exhibiting low binding energies and high intermolecular contact numbers. '+ 1', '+ 2', and '+ 3' denote number of other groups of eukaryotes a particular variant is found in (e.g., "Protists + 1" denotes a variant found in protists and one of the other groups—fungi, plants, or animals).

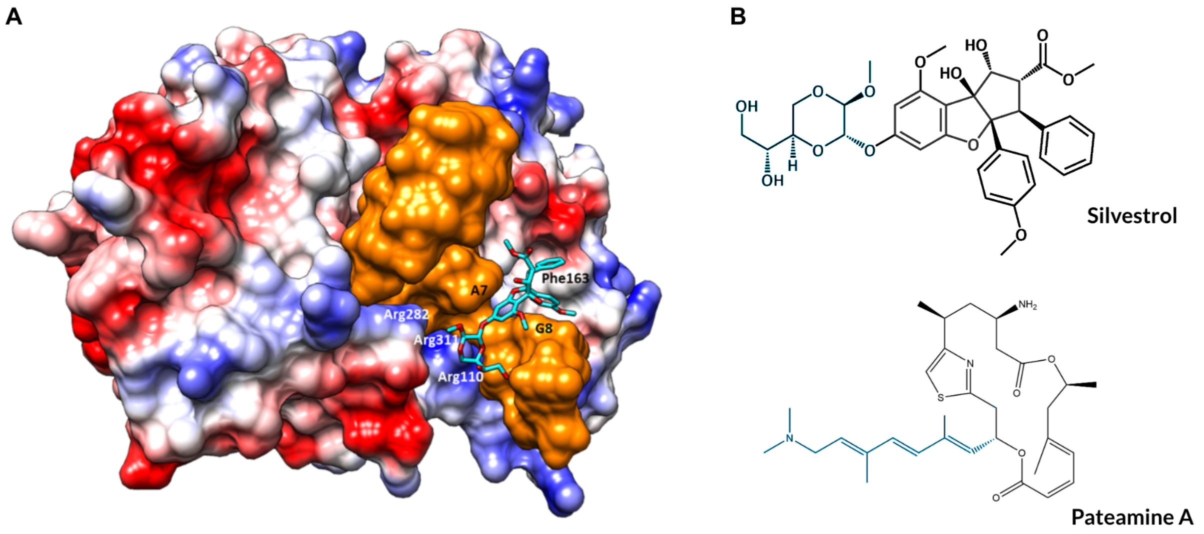

Figure S7: Expanded intermolecular contact interface between eIF4A and silvestrol or PatA. (**A**) Structure-based computational modeling of silvestrol onto the eIF4A-RNA complex (PDB: 5ZC9) illustrates how the 1,4-dioxane moiety of silvestrol can form additional contacts to those inside the RNA-binding pocket with three Arg residues on the surface of eIF4A—Arg110, Arg282, and Arg311—that form a highly conserved 'Arg pocket', resulting in a tighter clamp than the one generated by smaller rocaglates not having the 1,4-dioxane moiety. (**B**) Silvestrol and Pateamine A (PatA), a macrolide isolated from the marine sponge *Mycale hentscheli*, exhibit analogous interactions with this Arg pocket via their 1,4-dioxane and trienyl moieties, respectively (highlighted in blue) [53, 54]. PatA, which almost perfectly mimics the interaction of rocaglates with the RNA-binding pocket, also establishes hydrophobic interactions via its trienyl amine moiety with Arg110 and Arg 282 as well as with Gly304 and Asp305, two highly conserved residues involved in RNA-binding and closely associated with the QxxR motif [53]. This functional mimicry extends to the inability of PatA to stabilize the eIF4A:RNA complex in the presence of an F163L substitution [53, 55]. *Mycale hentscheli* is deeply rooted in the animal tree, one of the most recent branches of the Opisthokonta. The protein sequence of its eIF4A has not been determined, and no comprehensive map of eIF4A resistance/sensitivity to PatA exists, but potential resistance to PatA, based solely on the distribution of F163L mutations in eIF4A

determined by our analysis, is present in early, non-opisthokonta lineages that were presumably never exposed to PatA over evolutionary timescales, an intriguingly similar scenario to the one we describe for rocaglates. It would be interesting to next sequence eIF4A in *Mycale hentscheli* to determine whether it also contains resistant substitutions and which. (Electrostatic surface coloring of eIF4A generated with UCSF Chimera [blue: positively charged, red: negatively charged])

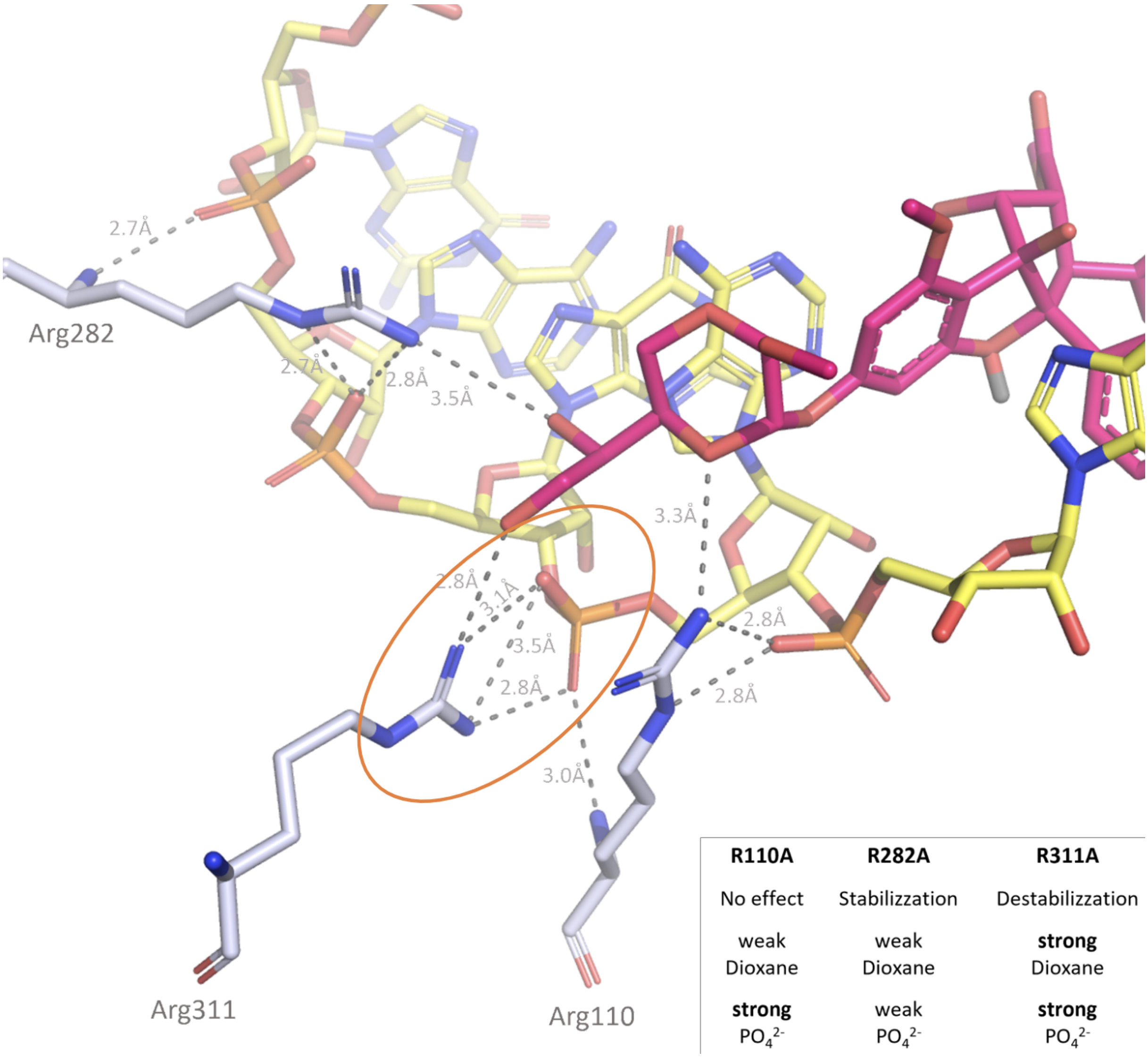

R110A	R282A	R311A
No effect	Stabilizzation	Destabilizzation
weak Dioxane	weak Dioxane	**strong** Dioxane
**strong** PO_4^{2-}	weak PO_4^{2-}	**strong** PO_4^{2-}

Figure S8: Structural interactions of the eIF4A:RNA:silvestrol complex in the Arg pocket of eIF4A. The dioxane moiety of silvestrol (magenta) interacts with the Arg rich pocket of eIF4A (grey stick) formed by Arg110, Arg282 and Arg311 (nitrogen atoms in blue and oxygen atoms in red). The Arg rich pocket is not only involved in a hydrogen bonding network (grey dashed lines) with silvestrol but it seems to play an important role also in the eIF4A-$(AG)_5$ complex formation. Based on both mutation and docking studies, we suggest the order of importance of Arg residues in RNA and silvestrol binding to be Arg311 > Arg110 > Arg282. Replacing these arginine key residues by alanine, the $eIF4A_{(19\text{-}406)}$:$(AG)_5$ complex cannot be formed efficiently or at all. Normally, rocaglates increase $eIF4A_{(19\text{-}406)}$:$(AG)_5$ complex stability by raising the T_m by 9.3°C (silvestrol), 8.4 °C (CR-1-31-B) and 8.0 °C (RocA). The R282A mutant shows also increased complex stability after addition of rocaglates (5,0 °C silvestrol, 3,9 °C CR-1-31-B and 3,6 °C RocA) when compared to the mutant protein alone, albeit to a lower extend (see Table S7). This may indicate that ternary complex formation is still possible but with somewhat reduced stability and is probably due to the weak interactions that Arg282 mediates with both the RNA A6 and the dioxane moiety. Therefore, this residue is important but not essential in the complex formation process. For the R110A mutant, there is virtually no difference in melting temperatures upon addition of the rocaglates (see Table S7). This suggests that rocaglates are not binding anymore to this mutant. Likely, R110 is involved in the $eIF4A_{(19\text{-}406)}$:$(AG)_5$ complex formation due to strong interaction with the phosphate group of RNA G8 which is not occurring in case of the Ala mutant. Moreover, Arg110 mediates only weak interactions with the dioxane moiety.

The most important residue of the series seems to be R311. When this residue is mutated to Ala, T_m is reduced upon addition of the rocaglates in a range between -3.1 and -6.3°C (see Table S7) indicating that the protein is evenly destabilized. Arg311 likely establishes a strong salt bridge (orange circle) with the phosphate groups of RNA A7 (yellow sticks with phosphorus atoms colored orange) and in a strong hydrogen bond with the dioxane moiety of silvestrol. An R311A mutant

precludes the formation of a stable eIF4A-RNA complex in the presence of rocaglates (see **inset**). Most likely, the equilibrium between complex formation and dissociation shifts toward dissociation because of the loss of the strong salt bridge with the RNA. Under these conditions, rocaglates cannot bind as well because the complex is probably not in the most favorable conformation to allow binding to occur. This is potentially the limiting step of complex formation: only when the RNA binds to eIF4A in the optimal conformation, the inhibitors can clamp and bind to the eIF4A-$(AG)_5$ complex, otherwise they probably bind more loosely, and they even destabilized the whole system. The behavior of the triple mutant is comparable with the single mutant R311A. Although no synergistic effect of the three mutated Arg residues is noted, a destabilizing effect with T_m reduction ranging from -3.2 °C to -3.6 °C is observed (see Table S7).

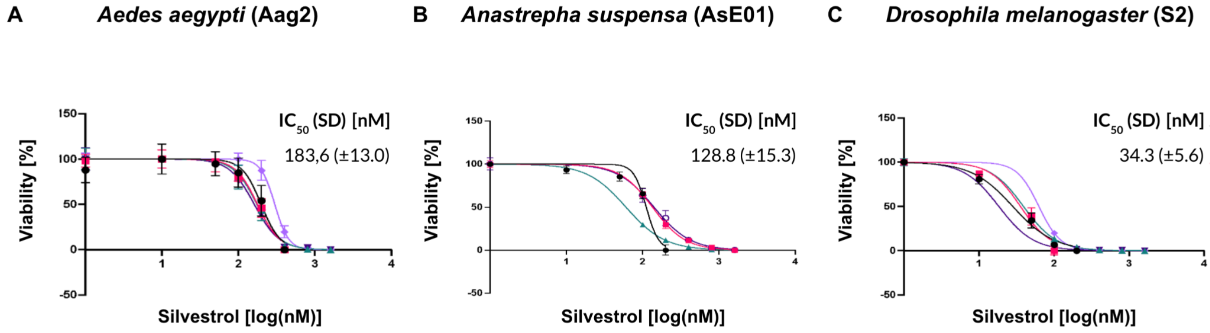

Figure S9: *In vitro* assays of sensitivity to silvestrol in mosquito and fruit fly cell lines expressing eIF4A proteins with previously untested and tested rocaglate-associated aa patterns. In all instances, viability was measured using a cell proliferation assay. (**A**) *Aedes aegypti* (Aag2) [TPHFQV; sensitive], (**B**) *Anastrepha suspensa* (AsE01) [presumably TPYFQV; sensitive], (**C**) *Drosophila melanogaster* (S2) [TPYFQV; sensitive].

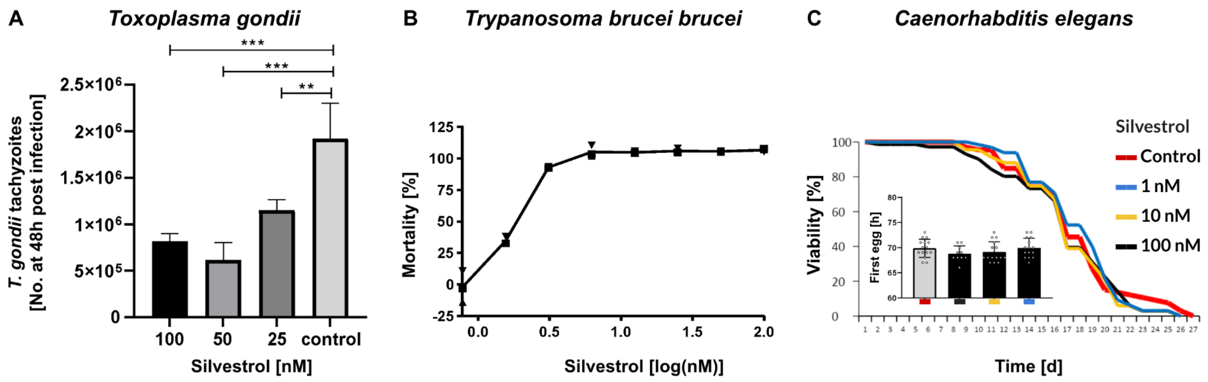

Figure S10: *In vitro* assays of sensitivity to silvestrol in organisms expressing eIF4A proteins with previously untested rocaglate-associated aa patterns. (**A**) *Toxoplasma gondii* [TPHFQV; sensitive] replication was measured using MARC145 monkey kidney cells as the host. (**B**) *Trypanosoma brucei brucei* [TPVFQI; sensitive] mortality was determined using a free parasite viability assay. (**C**) *Caenorhabditis elegans* [TPGFQV; resistant] viability and developmental pace (inset) were measured on plates.

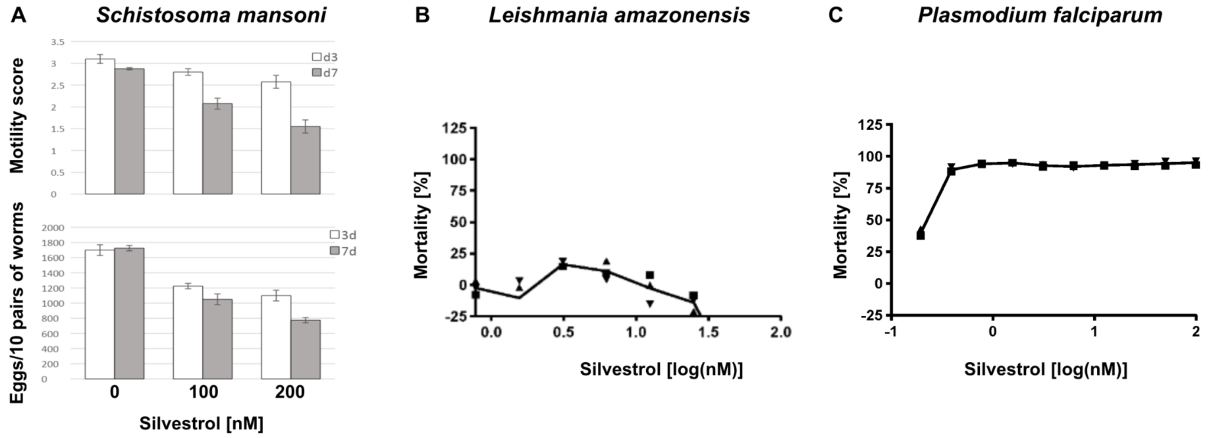

Figure S11: *In vitro* assays of sensitivity to silvestrol in organisms expressing eIF4A proteins with previously tested rocaglate-associated aa patterns. (**A**) *Schistosoma mansoni* [TPFFQI; sensitive] viability was measured using motility and egg production assays. (**B**) *Leishmania amazonensis* [TPSFQI; resistant] mortality was determined using a macrophage

infection assay. (**C**) *Plasmodium falciparum* [TPYFQV; sensitive] used a viability assay in erythrocytes.
